# Effects of growth hormone therapeutic supplementation on hematopoietic stem/progenitor cells in children with growth hormone deficiency: focus on proliferation and differentiation capabilities

**DOI:** 10.1007/s12020-015-0591-0

**Published:** 2015-04-29

**Authors:** M. P. Kawa, I. Stecewicz, K. Piecyk, E. Pius-Sadowska, E. Paczkowska, D. Rogińska, A. Sobuś, K. Łuczkowska, E. Gawrych, E. Petriczko, M. Walczak, B. Machaliński

**Affiliations:** Department of General Pathology, Pomeranian Medical University, Szczecin, Poland; Department of Pediatrics, Endocrinology, Diabetology, Metabolic Diseases and Cardiology of the Developmental Age, Pomeranian Medical University, Szczecin, Poland; Department of Pediatric and Oncological Surgery, Pomeranian Medical University, Szczecin, Poland

**Keywords:** Hematopoietic stem/progenitor cells, Hematopoiesis, Growth hormone deficiency, Growth hormone deficiency in children

## Abstract

We investigated the direct effects of growth hormone (GH) replacement therapy (GH-RT) on hematopoiesis in children with GH deficiency (GHD) with the special emphasis on proliferation and cell cycle regulation. Peripheral blood (PB) was collected from sixty control individuals and forty GHD children before GH-RT and in 3rd and 6th month of GH-RT to measure hematological parameters and isolate CD34^+^-enriched hematopoietic progenitor cells (HPCs). Selected parameters of PB were analyzed by hematological analyzer. Moreover, collected HPCs were used to analyze GH receptor (GHR) and IGF1 expression, clonogenicity, and cell cycle activity. Finally, global gene expression profile of collected HPCs was analyzed using genome-wide RNA microarrays. GHD resulted in a decrease in several hematological parameters related to RBCs and significantly diminished clonogenicity of erythroid progenies. In contrast, GH-RT stimulated increases in clonogenic growth of erythroid lineage and RBC counts as well as significant up-regulation of cell cycle-propagating genes, including *MAP2K1*, *cyclins D1/E1*, *PCNA,* and *IGF1*. Likewise, GH-RT significantly modified GHR expression in isolated HPCs and augmented systemic IGF1 levels. Global gene expression analysis revealed significantly higher expression of genes associated with cell cycle, proliferation, and differentiation in HPCs from GH-treated subjects. (i) GH-RT significantly augments cell cycle progression in HPCs and increases clonogenicity of erythroid progenitors; (ii) *GHR* expression in HPCs is modulated by GH status; (iii) molecular mechanisms by which GH influences hematopoiesis might provide a basis for designing therapeutic interventions for hematological complications related to GHD.

**Electronic supplementary material:**

The online version of this article (doi:10.1007/s12020-015-0591-0) contains supplementary material, which is available to authorized users.

## Introduction

Growth hormone (GH) is a pleiotropic hormone important for modulation of physiological processes [1]. Importantly, GH exerts its anabolic effects largely indirectly via stimulation of insulin-like growth factor-1 (IGF1) production. However, acting in autocrine/paracrine manner, GH binds to the specific receptor (GHR), which induces intracellular signaling pathways leading to differential gene expression [1]. The classical examples of IGF1-independent actions mediated through GHR include ovarian preantral follicle development [2] or activation of chondrocyte stem cells for chondrocyte generation at bone growth plate [3]. Similarly, in vitro studies indicate that neural stem cell activation by GH does not require IGF1 [4].

GH is also a potent modulator of hematopoietic system, which has been described in numerous experimental studies. GH binds to GHR expressed on hematopoietic/immune cells and regulates their proliferation and differentiation [5, 6]. GH was shown to augment in vitro erythropoiesis [7], granulopoiesis [8], and lymphopoiesis [9]. Likewise, supplementation with recombinant human GH (rhGH) was reported to reverse the age-induced hypocellularity of bone marrow in old rats [10]. rhGH administered to mice or non-human primates following lethal irradiation accelerated their hematopoietic recovery [11].

Isolated GHD (IGHD) is a rare disease and its final diagnosis is classically based on auxological and laboratory criteria, including short stature, reduced growth velocity, delayed bone age, and low GH responses to at least two pharmacological stimuli [12]. GHD patients exhibit numerous abnormalities in the hematological parameters of peripheral blood (PB), including microcytosis, anisocytosis, and poikilocytosis [13]. Furthermore, GHD patients commonly suffer from certain types of anemia, which are described in the literature as “IGHD-related anemia” [14]. The number of white blood cells is often reduced [15]. It was demonstrated that the metabolic abnormalities typical of GHD may be reversed by GH replacement therapy (GH-RT) [16]. However, the exogenous administration of rhGH, which has paracrine functions, does not necessarily mimic the physiological activity of GH, and the precise biological activity of rhGH requires further study. Data regarding rhGH-induced modulation of hematopoiesis during GH-RT are very limited, and only a few studies have evaluated the relationship between GHD and hematological parameters in children [17–20].

Therefore, we assessed the clinical outcomes of inappropriate GH signaling in CD34^+^-enriched hematopoietic stem and progenitor cells collected from IGHD children. In addition, we evaluated the effects of 6-month GH-RT on proliferation and differentiation of CD34^+^ cells and recovery of PB parameters.

## Materials and methods

### Subjects

We enrolled 40 children with severe IGHD, who were diagnosed according to the clinical criteria [12]. rhGH therapy was administered by subcutaneous injection at 0.031 mg/kg/d. None of the patients suffered from diabetes insipidus, chromosomal abnormalities, dysmorphic syndromes, intestinal malabsorption, other chronic diseases, or acquired GHD, as confirmed by a full clinical and laboratory evaluation. 60 children of similar ages, who did not differ significantly in terms of puberty and bone age, constituted the control group. All procedures were approved by local ethics committee, and informed consent was provided for each patient.

### Laboratory measurements and cell isolation

PB samples were collected at the moment of GHD diagnosis and after 3 and 6 months of GH-RT. We determined hematological and hormonal parameters and isolated CD34^+^-enriched hematopoietic progenitor cells (HPCs). The selected hematological parameters were evaluated using cell analyzer (Cell Dyn 3000, Abbott Diagnostics). The mononuclear cell fraction was isolated by density gradient centrifugation and depleted of adherent and T cells. This fraction was next enriched for CD34^+^ cells using the CD34 MicroBead Kit (Miltenyi Biotech, USA) according to the manufacturer’s protocol.

### RNA isolation and gene expression analysis

Total mRNA was isolated from HPCs using the RNeasy Mini Kit (Qiagen, USA). Subsequently, mRNA was reverse transcribed using the First Strand cDNA Synthesis Kit (Fermentas International Inc., Canada). Quantitative assessment of *GHR*, *MAP2K1*, *Cyclin D1*, *Cyclin E1*, *PCNA,* and *IGF1* mRNA levels was performed using real-time QRT-PCR carried out on a Bio-Rad CFX96 Real-Time PCR Detection System (Bio-Rad Inc., USA). The 25 µL reaction mixture contained 12.5 µL of SYBR Green PCR Master Mix, 10 ng of cDNA template, and one pair of the primers listed in Supplementary Table 1. The relative quantification value of the target gene was normalized to the endogenous control gene (BMG) and expressed as 2^ΔCt^, where ΔCt = [Ct of endogenous control gene] − [Ct of target gene].

### Flow cytometry

CD34^+^ HPCs and lymphocytes were analyzed with respect to the GHR expression. Briefly, erythrocytes in PB samples were lysed using BD PharmLyse Lysing Solution (BD Biosciences) for 15 min to obtain nucleated cells (NC). A total of 1 × 10^6^ NCs were incubated with mouse anti-human fluorochrome-conjugated monoclonal antibodies against specific antigens, including GHR, CD34, and CD45 (all from BD Biosciences) and analyzed by flow cytometry (LSRII, BD Biosciences). At least 0.5 × 10^6^ cells with the appropriate ratio of forward scatter to side scatter were acquired for the analysis.

### Clonogenic in vitro assays

CD34^+^ HPCs were evaluated using in vitro clonogenic assays. Briefly, 2 × 10^4^ cells were resuspended in 0.4 mL of RPMI-1640 medium (Sigma Aldrich, USA) and mixed with 1.8 mL of MethoCult HCC-4230 (StemCell Technologies Inc., Canada) supplemented with l-glutamine and antibiotics. To stimulate granulocyte–macrophage colony-forming units (CFU-GM), IL-3 (20 U/mL), SCF (10 ng/mL), and GM-CSF (5 ng/mL) were used. EPO (5 U/mL) and SCF (10 ng/mL) were used for induction of erythrocyte burst-forming units (BFU-E). IL-7 (5 ng/mL) and SCF (10 ng/mL) (all from R&D System) were used for induction of B-lymphocyte colony-forming units (CFU-B lymph). Each clonogenic test was performed in quadruplicate.

### Cell cycle analysis

Cell cycle progression in CD34^+^ HPCs was analyzed using the APO-Direct kit (BD Biosciences) according to the manufacturer’s instructions.

### ELISA

The systemic levels of IGF1 were measured using commercially available, high-sensitivity ELISA Quantikine human immunoassay kit (R&D Systems, USA) according to the manufacturer’s instructions.

### RNA isolation and Affymetrix GeneChip microarray and data analysis

Total RNA was isolated from CD34^+^ HPCs using RNeasy Mini Kit (Qiagen, USA). RNA was isolated from CD34^+^ cells of five GH-treated patients, the same at baseline, and in 3rd and 6th months of treatment, and of five control subjects, and was pooled to generate the final RNA sample representing a particular group in subsequent experimental procedures. Sense-strand cDNA generated from total RNA using an Ambion WT Expression Kit (Life Technologies, UK) was fragmented and labeled using the GeneChipH WT Terminal Labeling Kit (Affymetrix, USA) and hybridized onto an Affymetrix WT Array Strip. Hybridization as well as subsequent fluidics and scanning steps were performed using an Affymetrix GeneAtlasTM system (Affymetrix). Differences in the expression of the chosen genes and Gene Ontology (GO) terms were analyzed in the R programming environment using Bioconductor packages.

### Statistical methods

Differences in the values of the quantitative parameters were compared between groups by unpaired Student’s *t* test with Welch’s correction; for non-parametric tests, values were compared using the Mann–Whitney test. A *P* value of <0.05 was considered statistically significant.

## Results

### Characteristics of the clinical parameters

The characteristics of the subjects enrolled in the study are summarized in Table S2.

### Changes in the hematological parameters of patients with GHD

Selected PB parameters were measured at diagnosis and after 6 months of GH-RT as well in the control group (Table 1). Subjects were divided into two groups, 4–10 (“younger”) and 11–17 (“older”) years of age, due to common age-dependent changes in hematological parameters. We detected significantly decreased values of RBCs, HGB, and HCT, regardless of age, in untreated GHD patients compared to controls. Similarly, the GHD patients (11–17 years of age) exhibited significantly smaller MCH values, indicating a hypochromic state of RBCs. Moreover, GHD patients (4–10 years of age) exhibited significantly diminished MCV values, indicating microcytosis. In the same manner, we compared GHD patients before therapy and after 6 months of GH-RT and noticed a significant increase of RBCs, HGB, HCT, and MCV values after GH-RT, regardless of age. Finally, a statistically significant increase in MCH value was observed; however, this difference was only detected in younger GH-treated children. Of note, we were not able to detect any significant changes in platelets or WBCs in any of the analyzed groups. These data indicate that GH-RT could influence the morphological and functional changes in cells of erythropoietic lineage in GHD patients.

**Table 1:** Selected blood parameters in patients recruited for the study

Parameter	Age-matched GHD patients	GHD—before GH therapy Mean ± SD	GHD—6th month GH therapy Mean ± SD	Controls Mean ± SD
RBC (10^6^/mm^3^)	Younger	4,55 ± 0.08^#^	4.85 ± 0.08**	4.76 ± 0.06
	Older	4.58 ± 0.06^#^	4.86 ± 0.07**	4.86 ± 0.09
HGB (g/dl)	Younger	12.01 ± 0.26^#^	13.18 ± 0.23**	12.95 ± 0.23
	Older	12.86 ± 0.09^###^	13.88 ± 0.19****	13.91 ± 0.29
HCT (%)	Younger	35.29 ± 0.56^#^	38.52 ± 0.67***	37.26 ± 0.66
	Older	37.60 ± 0.43^###^	40.60 ± 0.56***	40.65 ± 0.70
MCV (mm^3^)	Younger	76.99 ± 0.58^##^	79.38 ± 0.84*	82.01 ± 1.79
	Older	81.42 ± 0.71	84.04 ± 0.84*	81.62 ± 1.05
MCH (pg)	Younger	26.30 ± 0.36	27.09 ± 0.26	27.63 ± 0.61
	Older	27.85 ± 0.31^#^	28.83 ± 0.31*	28.81 ± 0.34
MCHC (g/dl)	Younger	34.16 ± 0.32	34.21 ± 0.26	34.30 ± 0.44
	Older	34.25 ± 0.26	34.08 ± 0.23	34.06 ± 0.30
PLT (10^5^/mm^3^)	Younger	322.9 ± 11.89	313.8 ± 23.34	279.6 ± 17.30
	Older	285.5 ± 9.68	260.9 ± 8.35	254.3 ± 15.14
WBC (10^3^/mm^3^)	Younger	7.81 ± 0.51	7.853 ± 0.58	8.21 ± 0.68
	Older	6.386 ± 0.25	6.130 ± 0.23	7.131 ± 0.39

* P < 0.05; ** P < 0.01; *** *P* < 0.001; **** P < 0.0001 vs. values of GHD patients before therapy

^#^ *P* < 0.05; ^##^ *P* < 0.01; ^###^ *P* < 0.001 vs. controls

### *GHR* is expressed at the mRNA level in HPCs from GHD patients and is modulated by GH therapy

**Fig. 1:**
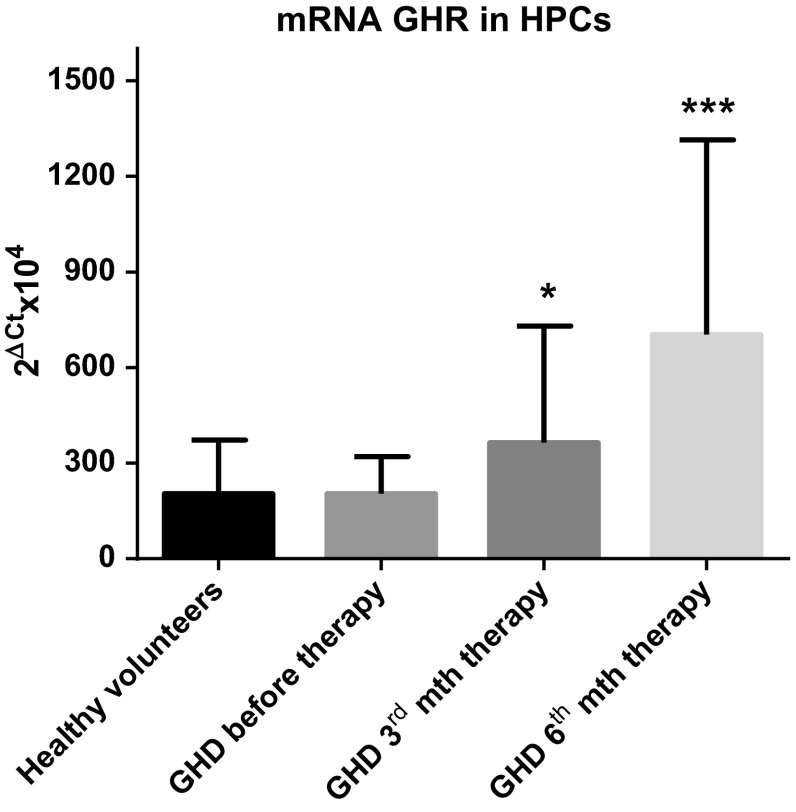
Expression of *GHR* mRNA in CD34^+^ HPCs of controls and GHD patients at different time points. Results are expressed as the mean value ± SD, *P < 0.05; ***P < 0.001 vs. GHD patients before therapy

Cells from all examined groups expressed *GHR* mRNA, We observed a strong around 300 % up-regulation of *GHR* mRNA in CD34^+^ HPCs collected after 6 months of GH-RT (*P* < 0.001; Fig. 1) and its brisk 50 % up-regulation after 3 months of GH-RT (*P* < 0.05; Fig. 1), compared to GHD patients before therapy. These data indicate that GHR expression could be influenced by GH therapy; thus, it may regulate GH-dependent effects in HPCs.

### The number of GHR^+^ hematopoietic cells increases during GH-RT in GHD patients

**Fig. 2:**
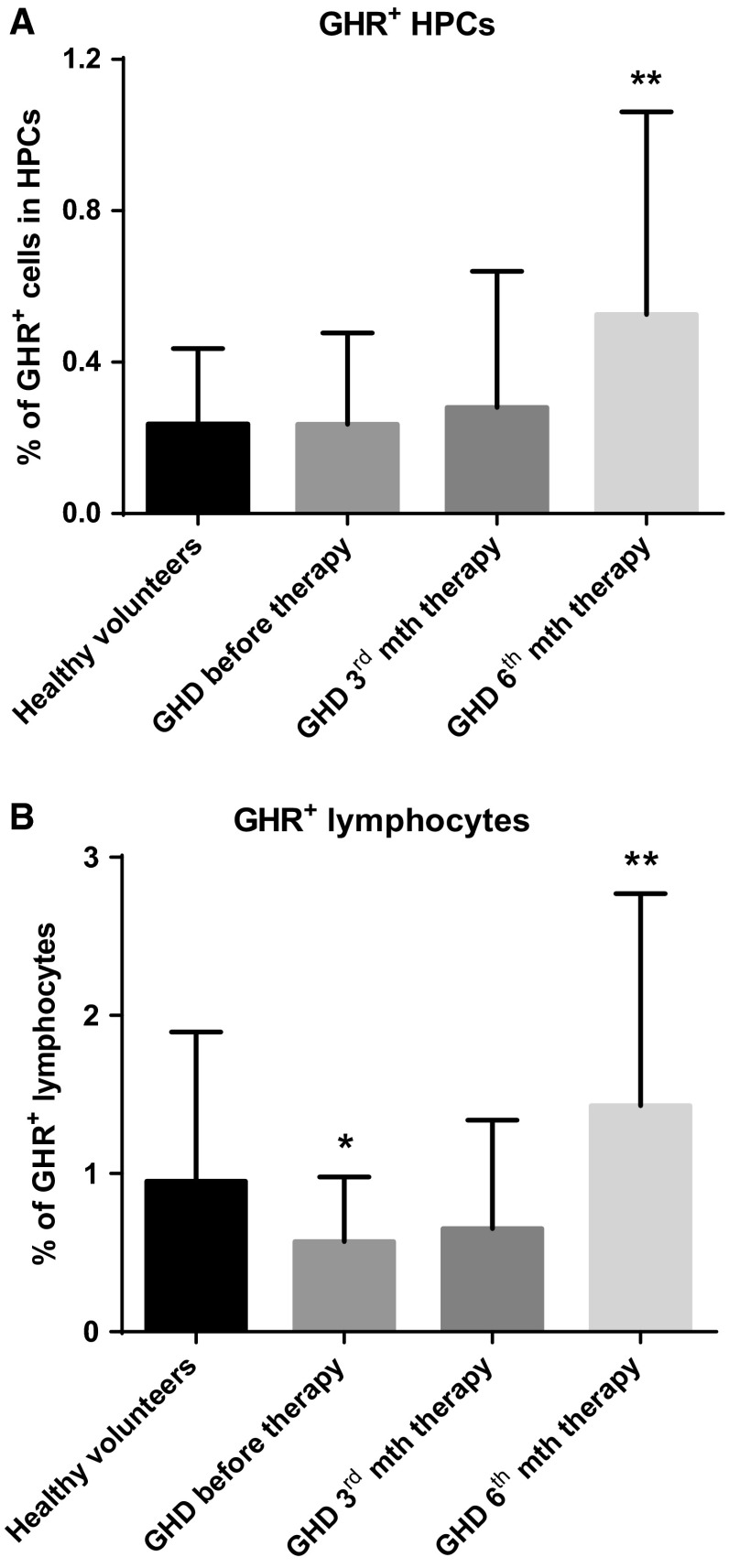
The percentage of GHR^+^ HPCs (**a**) and GHR^+^ lymphocytes (**b**) of controls and GHD patients at different time points. The results are expressed as the mean value ± SD. *P < 0.05; **P < 0.01 vs. GHD patients before therapy

Next, we investigated the relationship between GHR^+^ hematopoietic cell numbers and GH status. We simultaneously evaluated the number of GHR^+^ HPCs and GHR^+^ lymphocytes in PB before and during GH-RT (Fig. 2). We observed a significantly higher percentage of circulating GHR^+^ HPCs in patients after 6 months of GH-RT relative to GHD patients before therapy and controls (Fig. 2a). We also observed a slight, but insignificant, increase in the percentage of GHR^+^ HPCs in GHD patients after first 3 months of GH-RT (Fig. 2a). Similarly, we detected significantly increased percentage of GHR^+^ lymphocytes in GHD patients after 6 months of GH-RT relative to GHD patients before therapy (Fig. 2b); importantly, the latter group exhibited significantly decreased numbers of GHR^+^ lymphocytes compared to controls (Fig. 2b). The kinetics of the number of GHR^+^ HPCs detected during GH-RT largely mimicked the pattern of *GHR* mRNA expression detected in HPCs.

### Increased clonogenicity of BFU-E, but not CFU-GM or CFU-B lymph, from CD34^+^-enriched HPCs during the course of GH-RT

**Fig. 3:**
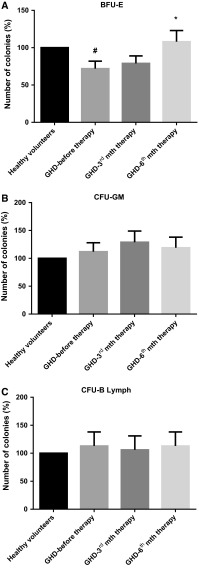
Comparative analysis of BFU-E (**a**), CFU-GM (**b**), and CFU-B lymph (**c**) clonogenicity of CD34^+^ HPCs from controls and GHD patients at different time points. The results are expressed as the percentage of the control value, which was set at 100 %. The results are presented as the mean value ± SD. *P < 0.05 vs. GHD patients before therapy; ^#^ *P* < 0.05 vs. control group

Subsequently, we examined the clonogenic potential of CD34^+^ HPCs in vitro. In GHD group, the number of CD34^+^-expanded erythroid BFU-E colonies was significantly lower than that observed in controls (72 vs. 100 %, respectively; *P* < 0.05; Fig. 3). Furthermore, we noted significantly increased clonogenicity of BFU-E colonies grown from the HPCs collected after 6 months of GH-RT, compared to those collected before GH-RT (108 vs. 72 %, respectively; *P* < 0.05; Fig. 3). These findings suggest that GH is involved in the systemic modulation of erythropoiesis in GHD patients. We also assessed the clonogenicity of CFU-GM and CFU-B lymph from HPCs collected from all groups, but observed no significant changes (Fig. 3b, c).

### GH-RT induces cell cycle progression in CD34^+^-enriched HPCs from GHD patients

**Fig. 4:**
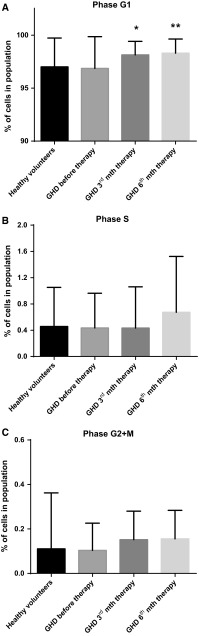
Analysis of the cell cycle phases (G1, S, and G2+M) in CD34^+^ HPCs of controls and GHD patients at different time points. The results are expressed as the mean value ± SD. *P < 0.05; **P < 0.01 vs. GHD patients before therapy

We also investigated whether GH may play a role in modulation of the cell cycle in HPCs following GH-RT. The cell cycle activity in HPCs was measured by the detection of cell cycle phases, i.e., the G1, S, and G2/M. We observed a significantly higher percentage of HPCs in the G1 phase from GHD patients after 3 (*P* < 0.05) and 6 (*P* < 0.01) months of GH-RT (Fig. 4a). GH treatment also generated an increase of approximately 50 % of the population of HPCs in the G2/M phase (3rd and 6th months of GH-RT) and S phase (6th month of GH-RT) compared to untreated patients (Fig. 4b, c). These data demonstrate the potential positive modulation by GH-RT of G1 phase in CD34^+^ cell cycle.

### GH-RT up-regulates proliferation-related gene expression in CD34^+^ HPCs in GHD patients

**Fig. 5:**
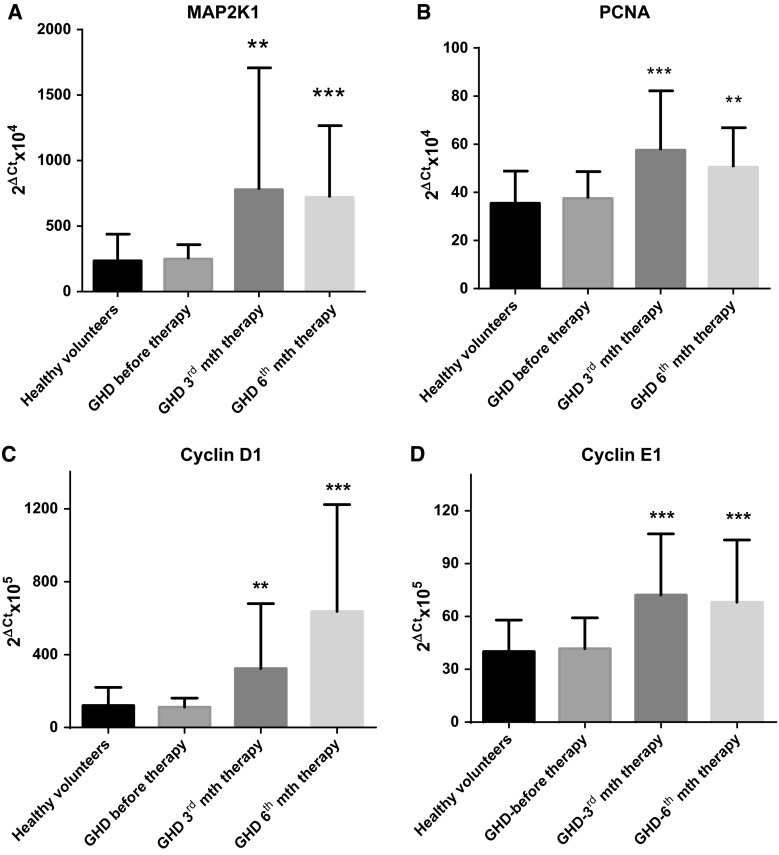
Expression of cell cycle-regulating genes in PB-derived CD34^+^ HPCs of controls and GHD patients at different time points. mRNA expression of the *MAP2K1* (**a**), *PCNA* (**b**), *CCND1* (**c**), and *CCNE1* (**d**) genes was determined. mRNA levels are expressed in arbitrary units as the mean value ± SD. **P < 0.01; ***P < 0.001 vs. GHD patients before therapy

GH promotes cell proliferation and differentiation. Therefore, we investigated whether GH-RT plays a role in HPC proliferation based on the analysis of the mRNA expression of genes crucial for the induction (*MAP2K1*) and maintenance (*PCNA*, *CCND1*, *CCNE1*) of cell proliferation. The mRNA levels for all of the examined genes were significantly higher in GHD patients after 3 and 6 months of GH-RT compared to GHD patients before therapy and controls. As shown in Fig. 5, there was around 300 % increase in the mRNA expression of *MAP2K1* and a similar increase in the mRNA expression of *CCND1*. Furthermore, we observed a nearly 100 % increase in *CCNE1* expression and a nearly 30 % increase in *PCNA* expression. These findings suggest that GH might be partially involved in the mechanisms responsible for induction of proliferation in HPCs from GHD patients treated with GH-RT.

### GH-RT modulates the IGF1 gene and protein expression in GHD patients

**Fig. 6:**
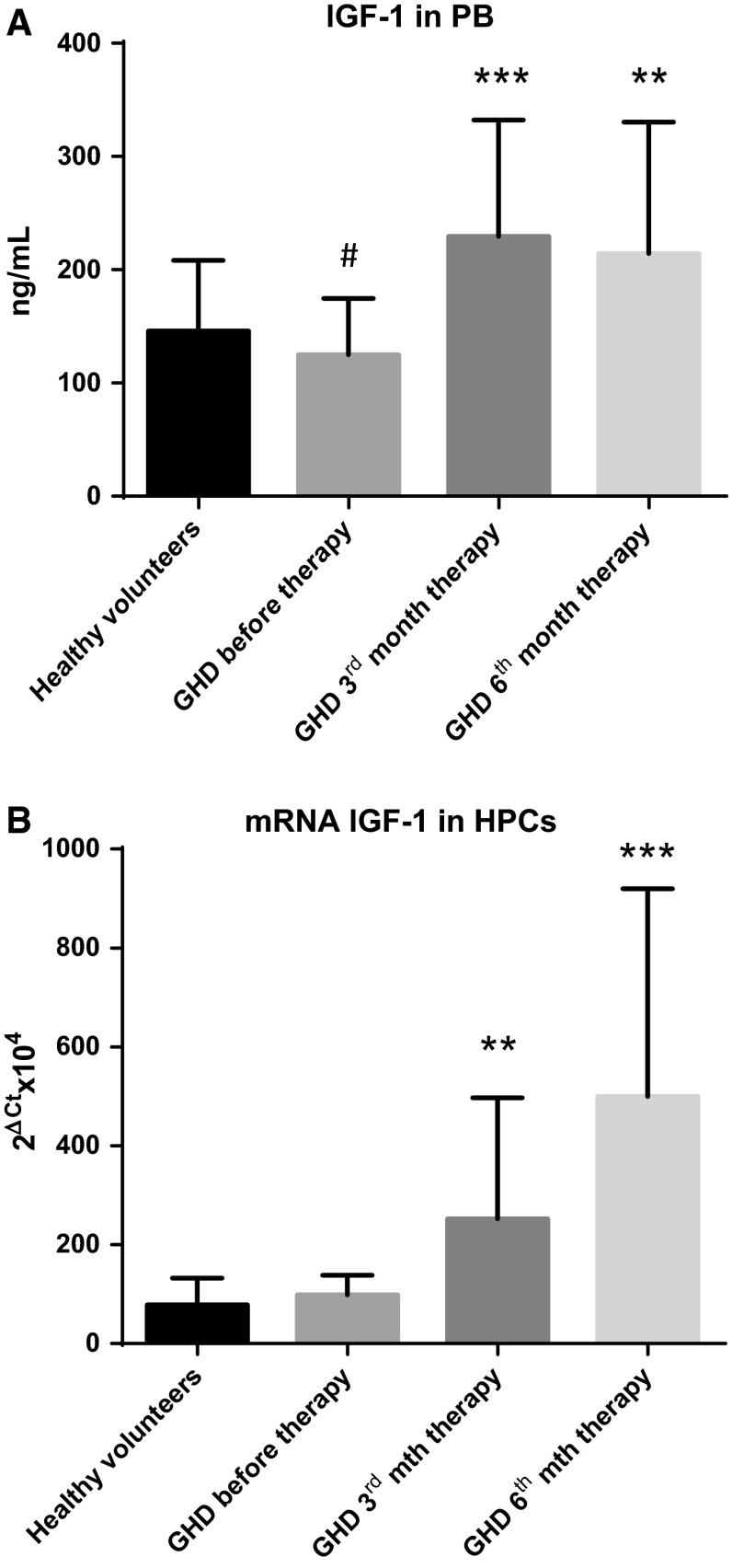
The levels of IGF1 in peripheral blood (**a**) and the expression of *IGF1* mRNA in CD34^+^ HPCs (**b**) of controls and GHD patients at different time points. The results are expressed as the mean value ± SD. **P < 0.01; ***P < 0.001 vs. GHD patients before therapy; ^#^ *P* < 0.05 vs. control group

Finally, we decided to verify potential association between GH therapy and expression of IGF1 in vivo. The mean systemic concentrations of IGF1 in GHD patients and control individuals are depicted in Fig. 6a. We found that the levels of IGF1 were significantly higher in GH-treated patients than those before therapy. Interestingly, systemic mean levels of IGF1 significantly differed between untreated GHD patients and their controls. Our data reveal that therapeutic intervention with rhGH provided to GHD patients could augment the global IGF1 production. In order to more extensively examine the expression of IGF1 in PB-derived CD34^+^ HPCs and to determine whether GH-RT could affect the *IGF1* gene transcription, we analyzed the levels of *IGF1* gene expression in HPCs collected from all examined groups. As shown in Fig. 6b, the mRNA expression of *IGF1* was significantly elevated in GHD patients during GH-RT compared to untreated patients and controls. No statistically significant differences in terms of *IGF1* mRNA levels were found in comparison of the untreated GHD patients and controls. These findings demonstrate that exogenous rhGH could trigger *IGF1* gene expression in CD34^+^ HPCs from GHD patients, which might have autocrine/paracrine impact on proliferation and survival of these cells.

### Whole-genome microarray analysis reveals large-scale alterations in gene expression in CD34^+^-enriched HPCs in GHD patients during the course of GH-RT

We analyzed the global gene expression pattern in CD34^+^-enriched HPCs collected from GHD patients before GH therapy and at selected time points (3 and 6 months post-GH-RT) and from controls.. In the GHD patients treated for 6 months with GH, we observed 177 genes that were up-regulated at least twofold (*P* < 0.05) (Table S3). The most significantly up-regulated gene in this group was the early activation antigen CD69 (*CD69*; 18-fold). CD69 is involved in WBC proliferation and is a signal-transmitting receptor in different subsets of WBCs. The other strongly up-regulated genes in this group included the regulator of G-protein signaling-1 (*RGS1*; 16-fold), which is involved in B-cell proliferation; asparaginyl-tRNA synthetase (*NARS*; tenfold), which belongs to the class II family of tRNA synthetases; radical S-adenosyl methionine domain containing 2 (*RSAD2*; ninefold), encoding viperin, which plays a role in CD4^+^ T cell activation and differentiation; and pleckstrin (*PLEK*; sevenfold), which is the platelet and leukocyte major protein C kinase substrate important for HPC differentiation. Of interest, our analysis identified several interleukins (IL) and related molecules (*IL1A*, *IL1B, IL8, ILF2, IL1RN, IL1R1, NFIL3,* and *IL5RA*), as well as chemokines (*CXCL1, CXCL2, CXCL16,* and *CCL4*) and chemokine receptors (*CXCR4, CXCR5, CCR4,* and *CCRL2*), as up-regulated after 6 months of GH-RT. Oncostatin M (*OSM*; fourfold), a growth regulator, was also found to be over-expressed. Furthermore, we found that several members of the zinc finger protein family, which act as transcriptional regulators involved in mitosis and cell development, including *EGR3, ZRANB2, ZNF146, RNF138, ZNF791*, and *ZNF766*, were significantly up-regulated after GH-RT. In contrast, in this group 21 genes were significantly down-regulated (Table S4). Similarly, we found that 3 months of GH treatment was responsible for significant up-regulation of 64 genes (Table S5) and down-regulation of 22 genes (Table S6). In addition, we found that prolongation of GH therapy from 3 to 6 months was responsible for significant up-regulation of 71 genes (Table S7) and down-regulation of 146 genes (Table S8).

We also analyzed the differences in gene expression profiles of untreated GHD patients compared to their healthy controls and observed the significant up-regulation of 16 genes (Table S9) and down-regulation of 129 genes (Table S10). Interestingly, untreated GHD patients exhibited significantly down-regulated expression of genes for several ILs and IL-related proteins (*IL1B, IL2RB, IL1R2, IL7R, ILF2*, and *ITK*) or chemokines and their receptors (*CXCL1, CCL4, CXCL16, CCR1, CXCR2,* and *CXCR1*). We also observed down-regulated genes involved in cell cycle control (*MAPK14, MDM2,* and *HCK*), which in general exhibit cytoprotective functions and are essential for cell survival.

Subsequently, 90 genes were specifically up-regulated at least twofold after 6-month GH-RT in GHD patients compared to their controls (Table S11). The genes mostly up-regulated during the course of GH-RT included interferon-induced protein 44-like (*IFI44L*; ninefold) and interferon-induced protein with tetratricopeptide repeats-1 (*IFIT1*; sevenfold), which are both members of the interferon-related immune response signaling pathway. Of importance, our analysis identified significant increases in the expression of essential regulators of cell cycle progression, such as *CDK6, SPIN1, TBL1XR1,JUN* and *APEX1*. Moreover, among the highly up-regulated genes, we observed those related to the positive regulation of hematopoiesis, including *MYB*, *HPGDS, LY6E,* and two markers of hematopoietic stem cells, CD133 (*PROM1*) and CD34. Furthermore, we found that, in the same period, the GH therapy induced the significant down-regulation of 45 genes (Table S12). In contrast, the 3-month-treatment with GH was responsible for significant up-regulation of 21 genes (Table S13) and down-regulation of 26 genes (Table S14).

**Fig. 7:**
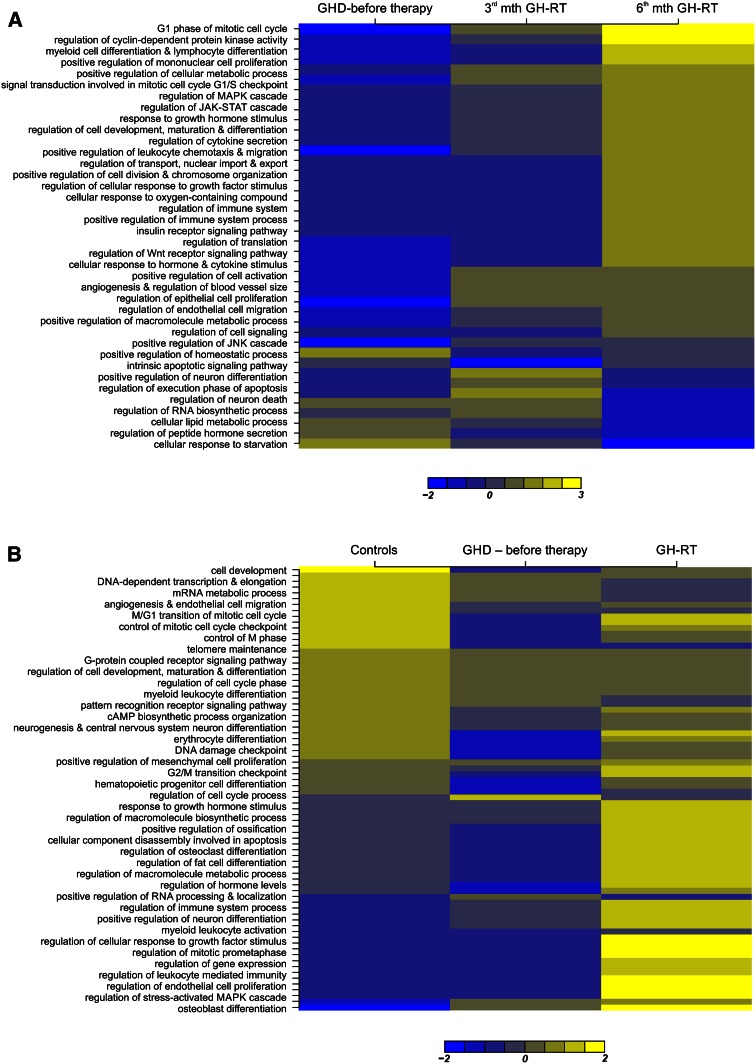
Global gene expression changes in CD34^+^ HPCs of controls and GHD patients at different time points (before GH-RT and in the 3rd and 6th months of GH-RT). The heatmap represents the expression levels of highly over-expressed genes (fold change >2). Individual genes are designated according to the GO classification of specific biological processes listed on the left side of the graph. Each *column* comprises a set of horizontal lines, each representing a single gene. The levels of gene expression are indicated on a color scale, with *yellow* corresponding to the highest level of expression and blue corresponding to the lowest level. The expression range of the analyzed genes is shown below the graph. The *upper diagram* (**a**) depicts large-scale alterations in gene expression between HPCs collected in the 3rd and 6th months of GH-RT compared to those collected before GH-RT. The *lower diagram* (**b**) presents large-scale alterations in gene expression between HPCs collected during the course of GH-RT compared to those collected before GH-RT as well as HPCs obtained from controls

Next, all of the differentially expressed genes were classified according to the GO classification of biological processes. Functional analysis using GO revealed that a number of pathways were specifically and diversely represented in the CD34^+^-enriched HPCs from GHD patients and controls. We observed that genes involved in the regulation of cell proliferation, regulation of cytokine production, signal transduction, regulation of angiogenesis and endothelial cell proliferation, positive regulation of immune system processes, regulation of gene expression, regulation of cell death, regulation of cellular metabolic process, regulation of homeostatic process, positive regulation of cellular processes, positive regulation of cytokine production, regulation of hormone transport and secretion, positive regulation of defense responses, regulation of responses to stress, wounding, and hypoxia were among the most up-regulated during GH-RT. A summary of the distribution of the selected gene clusters according to the GO classification results is presented in Fig. 7.

## Discussion

While the role of GH in hematopoiesis has been studied in several in vitro studies and animal models, the contribution of GH to hematopoiesis in GHD patients undergoing rhGH therapy remains incompletely understood. Although a number of GH-regulated genes have been identified, genes relating GH to the biology of HSPCs remain largely unknown. Accordingly, we sought to investigate the effects of 6-month GH-RT on cell cycle and HPCs proliferation in children with GHD. To our knowledge, the present study is the first to evaluate CD34^+^-enriched HPC functions with respect to GHD in children.

A considerable amount of evidence suggests that GH plays an important role in the modulation of hematopoiesis and exerts biological effects on erythropoiesis [7], granulopoiesis [8], and lymphopoiesis [21]. Importantly, the study of Sharma et al. on transgenic mice revealed that a genetic 100-fold reduction in GH expression did not affect hematopoietic cell expansion, maturation, or differentiation [22]. However, it is challenging to apply results obtained in vitro and in animal models to human physiology. As GHR expression is a key target that influences the effectiveness of GH, the analysis of its expression in HPCs is invaluable. The expression of GHR at the mRNA and protein levels was previously detected in human mature T and B lymphocytes and monocytes/macrophages [23, 24]. However, there is a lack of studies providing the evidence that GHR is expressed in CD34^+^-enriched HPCs from PB of GHD children. We observed in our study that GH-RT significantly augmented the basal expression levels of mRNA for *GHR*. Similarly, Hattori et al. showed previously a significant positive correlation between GH concentration and mRNA expression for *GHR* in normal human B lymphocytes cultured in vitro [23]. Lin et al. reported that, in mice bearing subcutaneous tumors naturally expressing GHR protein, the *GHR* mRNA expression was significantly increased after GH treatment [25]. These results indicate that the expression of *GHR* mRNA could be stimulated by increased GH concentrations. Indeed, we observed increased *GHR* mRNA expression and percentage of GHR^+^ HPCs in patients treated with GH-RT for 6 months. Similarly, the population of GHR^+^ lymphocytes was increased significantly (*P* < 0.01) after GH-RT. Furthermore, we observed that the quantity of GHR^+^ lymphocytes in untreated GHD patients was significantly decreased compared to that of controls. GH exerts effects on cell survival and proliferation also through its direct effects on several types of cells, including chondrocyte progenitors [26]. To further explore the potential direct GH influence on HPC proliferation and differentiation, we assessed the effect of GH-RT on HPCs clonogenicity. We demonstrated that clonogenic potential of CD34^+^-expanded BFU-E was significantly higher in GHD patients treated with GH-RT for 6 months than that before therapy. It is possible that this effect might be a result of the modulatory action of GH on the production of erythropoietin (EPO), as increased concentrations of GH augment the levels of EPO in GHD patients with developed anemia [27, 28], possibly due to increased hepatic synthesis of EPO [29]. However, in in vitro cultures containing an established amount of EPO, we observed increased growth only in the samples collected from patients treated previously with GH-RT. Indeed, previous studies have demonstrated that GH directly enhances the in vitro proliferation of erythroid precursors [7], and GH therapy had a stimulatory effect on the growth of BFU-E progenitors collected from 11 children with IGHD [30]. The observed significant increase in the reactivity of erythroid progenitors from GHD children during GH-RT may be the result of increased bioavailability of free, unbound GHR molecules on the progenitor cell surface. Additionally, GH may act as a potent direct cell growth stimulator, as shown in vitro using murine Friend erythroleukemia cells and human erythroleukemia cell line K562 [31, 32]. Taken together, these data suggest that the GH/GHR biological axis can directly induce signal transduction pathways involved in HSPC proliferation distinct from EPO-dependent pathways. This hypothesis is consistent with our another finding that CD34^+^ cells of GHD patients before therapy exhibited considerable growth reduction of BFU-E compared to those of the control subjects.

The GH-activated GHR induces pro-survival mechanisms downstream of the classical JAK-STAT pathway and finally initiates target gene transcription, which may result in increased stem cell cycling [33]. This potential mechanism of direct GH action in HPCs strongly correlates with our observations from the 3rd and 6th months of GH-RT, showing significantly increased numbers of CD34^+^ cells in G1 phase of the cell cycle, together with considerable increase of HPCs numbers in the S and G2+M phases, indicating that the number of proliferating cells might be increased during GH therapy. Likewise, GH simultaneously increased the mRNA expression of genes involved in cell cycle regulation, such as *MAP2K1*, cyclins *D1* and *E1*, and *PCNA*, in CD34^+^ HPCs from GH-treated individuals. These data correlate strongly with the changes in erythrocyte-related parameters including RBC, HCT, HGB, MCV, and MCH measured in the PB. Importantly, adult patients with GH deficiency have been characterized in several studies as anemic patients [30, 34]. In our study, at baseline the RBC numbers were decreased with reduced MCV and MCH values. In contrast, these values increased significantly within 6 months of GH-RT. Similarly, in the clinical study of GHD adults performed by Christ et al., the administration of GH led to an increase in RBC mass, indicating that GH may have a direct in vivo regulatory effect on erythropoiesis [35]. Additionally, Strauch et al. reported that, in acromegalic patients, RBC mass was increased but returned to normal values after curative surgery [36]. On the other hand, GHD patients suffer from significantly decreased HGB concentrations, regardless of their age. Subsequently, the HGB levels were significantly increased during GH-RT. Importantly, the final HGB concentration measured in GH-treated patients was maintained within the normal ranges and did not exhibit any superphysiological concentrations, as it has been reported after treatment with recombinant EPO [34]. Similarly, Miniero et al. evaluated 279 children with IGHD and diagnosed moderate anemia in 48 patients [14]. Moreover, the HGB levels significantly increased during GH-RT, and all examined children exhibited normal HGB values after 48 months of GH therapy [14]. GH is believed to increase the necessity for oxygen transport to the peripheral tissues due to its general anabolic effects, potentially resulting in increased HGB levels in PB. Together, these data support the concept that the GH/GHR axis directly promotes erythropoiesis in vivo in GHD children.

Although the liver is the major site of IGF-1 production (70 %) after stimulation by GH, IGF-1 is also produced locally by peripheral cells under basal conditions and in response to different stimuli [37]. Thus, local production of IGF1 also plays a role in the growth and differentiation of tissues, and several studies established the critical importance of IGF action for the development and normal function of hematopoietic system. It was demonstrated that IGF1 stimulates the growth, proliferation, and survival of different hematopoietic cell populations in vitro and inhibits their apoptosis [reviewed in detail in 38]. Moreover, Tsarfaty et al. observed that human IGF1 promotes hematopoietic growth in vivo in mice [15]. Likewise, GH and IGF1 administration enhanced reconstitution of the immune system and hematopoiesis after marrow transplantation in mice [39, 40]. Alpdogan et al. demonstrated that lymphoid and myeloid reconstitution after marrow transplantation was enhanced by IGF1 administration through the expansion of thymic precursor cell populations [41]. Vidal et al. reported that IGF1 was in the group of 52 acutely GH-activated genes in the liver 2 h after GH treatment [42]. Further analyses in mice with a targeted deletion of GHR have confirmed its key roles in controlling of IGF1 production [43]. In this notion, we conducted a preliminary analysis of the potential influence of GH-RT on *IGF1* mRNA expression in CD34 + HPCs from GHD patients and found that GH therapy significantly elevated its levels compared to GHD patients before therapy and controls. We also found the significantly higher IGF1 concentration in PB of GHD patients in course of GH-RT, which is in agreement with previous studies [44, 45]. From the molecular stand point, increased levels of IGF1 might be caused by several molecular mechanisms, which are GH dependent [46] and GH independent [47], with the liver as the main source of circulating hormone. However, our study demonstrates that CD34+HPCs can be a GH-dependent source of IGF1, which next may play pro-survival role and could potentially stimulate HPC proliferation in autocrine/paracrine manner. Moreover, we have found that exogenous GH is biologically active in CD34^+^ HPCs and stimulates the appropriate signal transduction pathways through GH/GHR signaling in these cells.

Finally, we have demonstrated for the first time that GH treatment is associated with specific changes in the global pattern of gene expression, which triggers diverse signaling pathways within HPCs from GHD children. A total of 177 genes were found to be differentially expressed after 6-month GH therapy compared to subjects before treatment. These genes were mainly associated with biological processes such as the regulation of cell cycle progression and mitosis, cell proliferation, hematopoietic and immune cell proliferation, maturation and differentiation, general cell development and differentiation, the regulation of translation, mRNA processing, cell migration and adhesion, cytokine secretion, intracellular and extracellular signaling pathways, cell metabolism, cell chemotaxis, angiogenesis, and apoptosis inhibition (Fig. 7a). Interestingly, our microarray experiments revealed that the three most strongly up-regulated genes were involved in hematopoietic T- and B-cell proliferation, activation, and differentiation. The above results could indicate that these GH-dependent changes in immune networks may improve immune functions and may therefore reverse some hematopoietic cell dysfunctions observed in GHD patients. Indeed, it has been observed that thymic function can be significantly enhanced by GH therapy [48]. Additionally, GH therapy was found to significantly increase the expression of several genes involved in signaling pathways of hematopoietic and immune cells, including cytokines (*IL1A, IL1B, IL8,* and *ILF2*) and chemokines (*CCL4, CXCL1, CXCL2,* and *CXCL16*), which are associated with cell proliferation and chemotaxis. Moreover, GH therapy induced the gene expression of tRNA synthetase, a crucial enzyme implicated in the synthesis of cellular components, i.e., nucleic acids, contributing in cell cycle control, mRNA transcription, splicing, and nuclear export of tRNAs [49]. Together, the above results imply that exogenous rhGH is likely to induce significant changes in cell cycle regulation, intracellular signaling, and cytokine production in HPCs from GHD children.

We further analyzed the influence of GH deficiency on gene expression changes in CD34^+^ HPCs. Specifically, the genes that were down-regulated due to GH deficiency could be broadly clustered into the following categories: cell cycle regulation and mitosis, hematopoietic cell lineage development and differentiation, cell chemotaxis, angiogenesis and endothelial cell migration, cytokine production and secretion, cytokine–cytokine receptor interaction, receptor signaling pathways, lipid metabolism, and biosynthesis of cell components (Fig. 7b). The most down-regulated gene in HPCs from GHD subjects was prostaglandin-endoperoxide synthase-2 (*PTGS2*), which acts both as a dioxygenase and a peroxidase, and thus is involved in the inflammation as well as the stimulation of mitosis [50]. Furthermore, the *c*-*MYB* gene encoding the proto-oncogene, which controls HSPC proliferation and differentiation [51], was also significantly down-regulated in GHD patients.. The above results demonstrate that GH deficiency can down-regulate the expression of genes that appear to be essential for cell cycle regulation, cell survival and proliferation, and differentiation of HPCs. Importantly, the changes in gene expression pattern found by our group in circulating HPCs from GHD children after 6-month GH therapy were also observed by other groups in mature PB-derived mononuclear cells from GHD children treated with GH for 1 or 3 months [52, 53]. In all analyses, GH treatment was associated with expression changes of genes involved in the processes such as cell function and metabolism, cell cycle, and cell migration. In particular, Stevens et al. observed that 1-month therapy with GH induced changes in MAPK- and SOS-mediated signaling pathways (e.g., IGF1) that are strongly related with cell growth and proliferation [52]. Similarly, 3-month GH treatment significantly induced expression of genes encoding regulators of G-protein signaling *(RGS1),* nuclear receptors *(NRF4A2),* and members of TNF-alpha family (TNFAIP3), as well as genes implicated in GH signaling, including *SOCS1* [53]. In addition, Fernandez-Perez et al. revealed that, among unique 163 genes differentially expressed in PB-derived mononuclear cells collected before and after 1-month of GH treatment in adult GHD patients, there were markedly expressed genes involved in SOCS-dependent intracellular signaling cascade (*ASB6*) or implicated in regulation of cell cycle progression (*PSMD8*), immune response (*IK*), signal transduction (*PABPN1*), or mRNA processing (*PPP2R5C*) [54]. Not surprisingly, the observed gene expression changes strongly involve the IGF1 pathway; therefore, the observed changes in gene expression in different cell populations circulating in PB could reflect both direct and indirect GH actions.

In conclusion, we report a direct cause and effect association between GHD and hematopoiesis in children. Our study demonstrates that CD34^+^ HPCs are sensitive to the pathophysiological conditions present in GHD and therapeutic GH replacement lasting at least 6 months. Our data also showed that GHD significantly impacts the proliferative potential of HPCs and their differentiation into erythroid-committed progenitors. Of note, we consider this work as an exploratory pilot study as the number of age- and sex-matched patients was small. Our observations may help improve the understanding of the interactions between rhGH and human hematopoiesis.

## Electronic supplementary material

Supplementary material 1 (DOC 117 kb)

Supplementary material 2 (DOC 1003 kb)
