# Supplementary material for: Effects of growth hormone therapeutic supplementation on hematopoietic stem/progenitor cells in children with growth hormone deficiency: focus on proliferation and differentiation capabilities
Source: Endocrine. 2015 Apr 29;50(1):162–75. doi: 10.1007/s12020-015-0591-0 (PMC4546702; doi:10.1007/s12020-015-0591-0)
Supplement: Supplementary file 1 — Supplementary material 1 (DOC 117 kb) [file 12020_2015_591_MOESM1_ESM.doc]

**Table S1:** List of primers used in this study

| No | Gene Name | Gene Symbol | Primer direction | Primer sequence |
| --- | --- | --- | --- | --- |
| 1 | growth hormone receptor | *GHR* | Sense | CCA TTG CCC TCA ACT GGA CTT |
|  |  |  | Antisense | AAT ATC TGC ATT GCG TGG TGC |
| 2 | cyclin D1 | *CCND1* | Sense | CGG TGT AGA TGC ACA GCT TCT C |
|  |  |  | Antisense | GCA TGT TCG TGG CCT CTA AGA T |
| 3 | cyclin E1 | *CCNE1* | Sense | ATC AGC ACT TTC TTG AGC AAC A |
|  |  |  | Antisense | TTG TGC CAA GTA AAA GGT CTC C |
| 4 | mitogen-activated protein kinase kinase 1 | *MAP2K1* | Sense | CAA TGG CGG TGT GGT GTT C |
|  |  |  | Antisense | AGC TCC CTT ATG ATC TGG TTC C |
| 5 | proliferating cell nuclear antigen | *PCNA* | Sense | TCC GCC ACC ATG TTC GA |
|  |  |  | Antisense | TAT CCC AGC AGG CCT CGT T |
| 6 | beta-2 macroglobulin | *BMG* | Sense | AAT GCG GCA TCT TCA AAC CT |
|  |  |  | Antisense | TGA CTT TGT CAC AGC CCA AGA TA |

| **Epidemiological and clinical**  **characteristics** | **GHD patients** | **Controls** |
| --- | --- | --- |
| Number of subjects/group (n) | 40 | 60 |
| Gender (male/female) (n) | 26/12 | 43/17 |
|  | **mean +/- SD** | **mean +/- SD** |
| Mean age (years) | 11 +/- 4.2 | 13 +/- 3.3 |
| Mean BMI | 18.65 +/- 3.5 | 19.17 +/- 3.1 |
| Plasma GH concentration (ng/mL) in different conditions: |  |  |
| 1. Mean nocturnal GH release | 5.6 +/- 2.7 | NA |
| 2. The oral clonidine provocative test | 5.7 +/- 2.4 | NA |
| 3. The L-Dopa provocative test | 4.5 +/- 2.2 | NA |
| Plasma IGF-1 concentration (ng/mL) | 125 +/- 49.6 | 162.3 +/- 58.6 |
| Plasma IGF-BP-3 concentration (µg/mL) | 4.0 +/- 1.6 | 4.15 +/- 0.86 |
| Number of subjects in “younger” group (n) | 17 | 23 |
| Number of subjects in “older” group (n) | 23 | 37 |
| Mean age of “younger” group (years) | 6.7 +/- 2,5 | 8.9 +/- 1,4 |
| Mean age of “older” group (years) | 13.7 +/- 1.8 | 14.3 +/- 1,9 |
| Mean BMI of “younger” group | 16.79 +/- 1.9 | 16.94 +/- 0.7 |
| Mean BMI of “older” group | 19.83 +/- 3.8 | 20.6 +/- 3.1 |

**Table S2:** Clinical characteristics of the study population
