# Supplementary material for: Effects of growth hormone therapeutic supplementation on hematopoietic stem/progenitor cells in children with growth hormone deficiency: focus on proliferation and differentiation capabilities
Source: Endocrine. 2015 Apr 29;50(1):162–75. doi: 10.1007/s12020-015-0591-0 (PMC4546702; doi:10.1007/s12020-015-0591-0)
Supplement: Supplementary file 2 — Supplementary material 2 (DOC 1003 kb) [file 12020_2015_591_MOESM2_ESM.doc]

**Supplementary Table S3.** The up-regulated genes with the largest significant change of expression (at least 2-fold) in CD34^+^-enriched HPCs from GHD patients treated for 6 months with GH-RT compared to GHD patients before therapy

| No | Gene Symbol | log_2_(6mth GH-RT /untreated GHD) | Gene Name |
| --- | --- | --- | --- |
| 1 | CD69 | 4,17 | CD69 molecule |
| 2 | RGS1 | 3,98 | regulator of G-protein signaling 1 |
| 3 | NARS | 3,28 | asparaginyl-tRNA synthetase |
| 4 | RSAD2 | 3,14 | radical S-adenosyl methionine domain containing 2 |
| 5 | PLEK | 2,84 | pleckstrin |
| 6 | IFI44L | 2,48 | interferon-induced protein 44-like |
| 7 | IFI44 | 2,42 | interferon-induced protein 44 |
| 8 | OLR1 | 2,35 | oxidized low density lipoprotein (lectin-like) receptor 1 |
| 9 | EIF3D | 2,34 | eukaryotic translation initiation factor 3, subunit D |
| 10 | HNRNPC | 2,33 | heterogeneous nuclear ribonucleoprotein C (C1/C2) |
| 11 | DNAJB1 | 2,15 | DnaJ (Hsp40) homolog, subfamily B, member 1 |
| 12 | ARL5B | 2,10 | ADP-ribosylation factor-like 5B |
| 13 | OSM | 2,07 | oncostatin M |
| 14 | HES1 | 2,06 | hairy and enhancer of split 1, (Drosophila) |
| 15 | IFIT3 | 2,04 | interferon-induced protein with tetratricopeptide repeats 3 |
| 16 | FOSB | 2,00 | FBJ murine osteosarcoma viral oncogene homolog B |
| 17 | HBEGF | 1,96 | heparin-binding EGF-like growth factor |
| 18 | KAT7 | 1,91 | K(lysine) acetyltransferase 7 |
| 19 | COX7B | 1,91 | cytochrome c oxidase subunit VIIb |
| 20 | TNFAIP3 | 1,86 | tumor necrosis factor, alpha-induced protein 3 |
| 21 | HCAR2 | 1,86 | hydroxycarboxylic acid receptor 2 |
| 22 | ILF2 | 1,85 | interleukin enhancer binding factor 2, 45kDa |
| 23 | NR4A2 | 1,85 | nuclear receptor subfamily 4, group A, member 2 |
| 24 | TARDBP | 1,81 | TAR DNA binding protein |
| 25 | HERC5 | 1,81 | HECT and RLD domain containing E3 ubiquitin protein ligase 5 |
| 26 | PSMB2 | 1,79 | proteasome (prosome, macropain) subunit, beta type, 2 |
| 27 | IL1RN | 1,78 | interleukin 1 receptor antagonist |
| 28 | IL1A | 1,74 | interleukin 1, alpha |
| 29 | IL1B | 1,73 | interleukin 1, beta |
| 30 | CCRL2 | 1,73 | chemokine (C-C motif) receptor-like 2 |
| 31 | PRPF8 | 1,72 | PRP8 pre-mRNA processing factor 8 homolog |
| 32 | CBX3 | 1,69 | chromobox homolog 3 |
| 33 | ICAM1 | 1,68 | intercellular adhesion molecule 1 |
| 34 | CXCL1 | 1,65 | chemokine (C-X-C motif) ligand 1 |
| 35 | ABCF1 | 1,65 | ATP-binding cassette, sub-family F (GCN20), member 1 |
| 36 | IFIT2 | 1,65 | interferon-induced protein with tetratricopeptides 2 |
| 37 | TNFAIP6 | 1,64 | tumor necrosis factor, alpha-induced protein 6 |
| 38 | OAS3 | 1,62 | 2'-5'-oligoadenylate synthetase 3, 100kDa |
| 39 | RNPS1 | 1,62 | RNA binding protein S1, serine-rich domain |
| 40 | JTB | 1,61 | jumping translocation breakpoint |
| 41 | THBS1 | 1,59 | thrombospondin 1 |
| 42 | PTGS2 | 1,58 | prostaglandin-endoperoxide synthase 2 (prostaglandin G/H synthase and cyclooxygenase) |
| 43 | PFKFB3 | 1,56 | 6-phosphofructo-2-kinase/fructose-2,6-biphosphatase 3 |
| 44 | IER3 | 1,55 | immediate early response 3 |
| 45 | B3GNT5 | 1,53 | beta-1,3-N-acetylglucosaminyltransferase 5 |
| 46 | IFI6 | 1,53 | interferon, alpha-inducible protein 6 |
| 47 | snoU13 | 1,53 | Small nucleolar RNA U13 |
| 48 | HCAR3 | 1,52 | hydroxycarboxylic acid receptor 3 |
| 49 | NR4A3 | 1,51 | nuclear receptor subfamily 4, group A, member 3 |
| 50 | NFIL3 | 1,49 | nuclear factor, interleukin 3 regulated |
| 51 | GLA | 1,48 | galactosidase, alpha |
| 52 | SNORA51 | 1,47 | Small nucleolar RNA SNORA51 |
| 53 | SOCS3 | 1,47 | suppressor of cytokine signaling 3 |
| 54 | SART3 | 1,47 | squamous cell carcinoma antigen recognized by T cells 3 |
| 55 | PER1 | 1,46 | period homolog 1 |
| 56 | LIPN | 1,46 | lipase, family member N |
| 57 | SYAP1 | 1,43 | synapse associated protein 1 |
| 58 | IGJ | 1,40 | immunoglobulin J polypeptide, linker protein for immunoglobulin alpha and mu polypeptides |
| 59 | IFNG | 1,39 | interferon, gamma |
| 60 | HINT1 | 1,39 | histidine triad nucleotide binding protein 1 |
| 61 | PPIF | 1,36 | peptidylprolyl isomerase F |
| 62 | MIR877 | 1,36 | microRNA 877 |
| 63 | CD83 | 1,36 | CD83 molecule |
| 64 | RHOA | 1,36 | ras homolog family member A |
| 65 | IFIT5 | 1,36 | interferon-induced protein with tetratricopeptide repeats 5 |
| 66 | MX1 | 1,35 | myxovirus (influenza virus) resistance 1, interferon-inducible protein p78 (mouse) [Source:HGNC Symbol |
| 67 | CMPK2 | 1,35 | cytidine monophosphate (UMP-CMP) kinase 2, |
| 68 | CXCL16 | 1,34 | chemokine (C-X-C motif) ligand 16 |
| 69 | SNAI1 | 1,31 | snail homolog 1 |
| 70 | OASL | 1,30 | 2'-5'-oligoadenylate synthetase-like |
| 71 | CCL4 | 1,29 | chemokine (C-C motif) ligand 4 |
| 72 | PTGES3P1 | 1,29 | prostaglandin E synthase 3 (cytosolic) pseudogene 1 |
| 73 | NFKBID | 1,28 | nuclear factor of kappa light polypeptide gene enhancer in B-cells inhibitor, delta |
| 74 | FUNDC2 | 1,28 | FUN14 domain containing 2 |
| 75 | ATP6V0B | 1,28 | ATPase, lysosomal 21kDa, V0 subunit b |
| 76 | CTDNEP1 | 1,27 | CTD nuclear envelope phosphatase 1 |
| 77 | EIF2AK2 | 1,27 | eukaryotic translation initiation factor 2-alpha kinase 2 |
| 78 | BCAS2 | 1,26 | breast carcinoma amplified sequence 2 |
| 79 | ATP6V1D | 1,26 | ATPase, lysosomal 34kDa, V1 subunit D |
| 80 | CXCL2 | 1,26 | chemokine (C-X-C motif) ligand 2 |
| 81 | TMEM30A | 1,25 | transmembrane protein 30A |
| 82 | CFL2 | 1,25 | cofilin 2 (muscle) |
| 83 | FCAR | 1,23 | Fc fragment of IgA, receptor |
| 84 | JOSD1 | 1,22 | Josephin domain containing 1 |
| 85 | COQ10B | 1,22 | coenzyme Q10 homolog B |
| 86 | HSPH1 | 1,22 | heat shock 105kDa/110kDa protein 1 |
| 87 | GPR183 | 1,22 | G protein-coupled receptor 183 |
| 88 | RASGEF1B | 1,22 | RasGEF domain family, member 1B |
| 89 | NR4A1 | 1,21 | nuclear receptor subfamily 4, group A, member 1 |
| 90 | SERPING1 | 1,21 | serpin peptidase inhibitor, clade G, member 1 |
| 91 | EGR3 | 1,19 | early growth response 3 |
| 92 | PMAIP1 | 1,19 | phorbol-12-myristate-13-acetate-induced protein 1 |
| 93 | PLAUR | 1,19 | plasminogen activator, urokinase receptor |
| 94 | LAMTOR3 | 1,18 | late endosomal/lysosomal adaptor, MAPK/MTOR activator 3 |
| 95 | CSRNP1 | 1,17 | cysteine-serine-rich nuclear protein 1 |
| 96 | CXCR4 | 1,16 | chemokine (C-X-C motif) receptor 4 |
| 97 | MAFF | 1,16 | v-maf musculoaponeurotic fibrosarcoma oncogene homolog F |
| 98 | SIK1 | 1,16 | salt-inducible kinase 1 |
| 99 | PLSCR1 | 1,15 | phospholipid scramblase 1 |
| 100 | SNORA40 | 1,15 | Small nucleolar RNA SNORA40 |
| 101 | PLK3 | 1,14 | polo-like kinase 3 |
| 102 | JUN | 1,14 | jun proto-oncogene |
| 103 | ARF1 | 1,14 | ADP-ribosylation factor 1 |
| 104 | OCLM | 1,14 | oculomedin |
| 105 | RPL11 | 1,14 | ribosomal protein L11 |
| 106 | IFIH1 | 1,13 | interferon induced with helicase C domain 1 |
| 107 | EIF3F | 1,13 | eukaryotic translation initiation factor 3, subunit F |
| 108 | DCUN1D1 | 1,13 | DCN1, defective in cullin neddylation 1, domain containing 1 |
| 109 | TAF10 | 1,13 | TAF10 RNA polymerase II, TATA box binding protein (TBP)-associated factor, 30kDa |
| 110 | CHMP2B | 1,13 | charged multivesicular body protein 2B |
| 111 | RPS3AP5 | 1,12 | ribosomal protein S3A pseudogene 5 |
| 112 | ZRANB2 | 1,12 | zinc finger, RAN-binding domain containing 2 |
| 113 | IVNS1ABP | 1,11 | influenza virus NS1A binding protein |
| 114 | AP3S1 | 1,11 | adaptor-related protein complex 3, sigma 1 subunit |
| 115 | RIPK2 | 1,11 | receptor-interacting serine-threonine kinase 2 |
| 116 | DDX60L | 1,10 | DEAD (Asp-Glu-Ala-Asp) box polypeptide 60-like |
| 117 | RNF138 | 1,10 | ring finger protein 138, E3 ubiquitin protein ligase |
| 118 | NFKBIZ | 1,09 | nuclear factor of kappa light polypeptide gene enhancer in B-cells inhibitor, zeta |
| 119 | NMI | 1,09 | N-myc (and STAT) interactor |
| 120 | HIST1H2BK | 1,09 | histone cluster 1, H2bk |
| 121 | TRIB1 | 1,08 | tribbles homolog 1 (Drosophila) |
| 122 | TMEM106B | 1,08 | transmembrane protein 106B |
| 123 | KXD1 | 1,08 | KxDL motif containing 1 |
| 124 | DUSP10 | 1,07 | dual specificity phosphatase 10 |
| 125 | SULT1B1 | 1,07 | sulfotransferase family, cytosolic, 1B, member 1 |
| 126 | NMD3 | 1,06 | NMD3 homolog |
| 127 | MTND4P9 | 1,06 | MT-ND4 pseudogene 9 |
| 128 | ZNF791 | 1,06 | zinc finger protein 791 |
| 129 | PPP1R15A | 1,05 | protein phosphatase 1, regulatory subunit 15A |
| 130 | MT2A | 1,05 | metallothionein 2A |
| 131 | BNIP2 | 1,04 | BCL2/adenovirus E1B 19kDa interacting protein 2 |
| 132 | SRSF9 | 1,04 | serine/arginine-rich splicing factor 9 |
| 133 | MORF4L2 | 1,04 | mortality factor 4 like 2 |
| 134 | DNAJB4 | 1,04 | DnaJ (Hsp40) homolog, subfamily B, member 4 |
| 135 | IMPAD1 | 1,04 | inositol monophosphatase domain containing 1 |
| 136 | TREM1 | 1,04 | triggering receptor expressed on myeloid cells 1 |
| 137 | OAS1 | 1,03 | 2'-5'-oligoadenylate synthetase 1, 40/46kDa |
| 138 | SCML1 | 1,03 | sex comb on midleg-like 1 |
| 139 | IFT20 | 1,03 | Intra-flagellar transport 20 homolog |
| 140 | GSTM4 | 1,03 | glutathione S-transferase mu 4 |
| 141 | FTH1P11 | 1,03 | ferritin, heavy polypeptide 1 pseudogene 11 |
| 142 | CCDC125 | 1,03 | coiled-coil domain containing 125 |
| 143 | SLC7A5 | 1,03 | solute carrier family 7 (amino acid transporter light chain, L system), member 5 |
| 144 | RBMS1 | 1,03 | RNA binding motif, single stranded interacting protein 1 |
| 145 | PARK7 | 1,02 | parkinson protein 7 |
| 146 | DAD1 | 1,02 | defender against cell death 1 |
| 147 | ATF3 | 1,02 | activating transcription factor 3 |
| 148 | ATP5B | 1,02 | ATP synthase, H+ transporting, mitochondrial F1 complex, beta polypeptide |
| 149 | SESN2 | 1,02 | sestrin 2 |
| 150 | ETF1 | 1,02 | eukaryotic translation termination factor 1 |
| 151 | SEPT2 | 1,02 | septin 2 |
| 152 | RRAGD | 1,02 | Ras-related GTP binding D |
| 153 | CWC25 | 1,02 | CWC25 spliceosome-associated protein |
| 154 | UGCG | 1,02 | UDP-glucose ceramide glucosyltransferase |
| 155 | LY96 | 1,02 | lymphocyte antigen 96 |
| 156 | PLIN2 | 1,01 | perilipin 2 |
| 157 | OXR1 | 1,0 | oxidation resistance 1 |
| 158 | AAED1 | 1,0 | AhpC/TSA antioxidant enzyme domain containing 1 |
| 159 | FAM91A1 | 1,0 | family with sequence similarity 91, member A1 |
| 160 | XAF1 | 1,0 | XIAP associated factor 1 |
| 161 | INSIG1 | 1,0 | insulin induced gene 1 |
| 162 | RNU4-9P | 1,0 | RNA, U4 small nuclear 9, |
| 163 | ZNF146 | 1,0 | zinc finger protein 146 |
| 164 | FBXO33 | 1,0 | F-box protein 33 |
| 165 | COG6 | 1,0 | component of oligomeric golgi complex 6 |
| 166 | DDIT4 | 1,0 | DNA-damage-inducible transcript 4 |
| 167 | VEGFA | 1,0 | vascular endothelial growth factor A |
| 168 | DNTTIP2 | 1,0 | deoxynucleotidyltransferase, interacting protein 2 |
| 169 | PTP4A1P7 | 1,0 | protein tyrosine phosphatase IVA, member 1 pseudogene 7 |
| 170 | PGAP2 | 1,0 | post-GPI attachment to proteins 2 |
| 171 | GNAI3 | 1,0 | guanine nucleotide binding protein (G protein), alpha inhibiting activity polypeptide 3 |
| 172 | BHLHE40 | 1,0 | basic helix-loop-helix family, member e40 |
| 173 | CDKN1A | 1,0 | cyclin-dependent kinase inhibitor 1A (p21, Cip1) |
| 174 | SNRPA1 | 1,0 | small nuclear ribonucleoprotein polypeptide A' |
| 175 | HSDL2 | 1,0 | hydroxysteroid dehydrogenase like 2 |
| 176 | PNPLA8 | 1,0 | patatin-like phospholipase domain containing 8 |
| 177 | GATC | 1,0 | glutamyl-tRNA(Gln) amidotransferase, subunit C |

**Supplementary Table S4.** The down-regulated genes with the largest significant change of expression (at least 2-fold) in CD34^+^-enriched HPCs from GHD patients treated for 6 months with GH-RT compared to GHD patients before therapy

| No | Gene Symbol | log_2_(6mth GH-RT /untreated GHD) | Gene Name |
| --- | --- | --- | --- |
| 1 | FNTA | 2,8 | farnesyltransferase, CAAX box, alpha |
| 2 | RNU5E-1 | 1,9 | RNA, U5E small nuclear 1 |
| 3 | SMNDC1 | 1,9 | survival motor neuron domain containing 1 |
| 4 | SNRNP200 | 1,8 | small nuclear ribonucleoprotein 200kDa (U5) |
| 5 | SART1 | 1,6 | squamous cell carcinoma antigen recognized by T cells |
| 6 | RNU12 | 1,5 | RNA, U12 small nuclear |
| 7 | RNA5SP415 | 1,2 | RNA, 5S ribosomal pseudogene 415 |
| 8 | CAPNS1 | 1,2 | calpain, small subunit 1 |
| 9 | RNY3P3 | 1,2 | RNA, Ro-associated Y3 pseudogene 3 |
| 10 | NPPC | 1,2 | natriuretic peptide C |
| 11 | RNU4-1 | 1,2 | RNA, U4 small nuclear 1 |
| 12 | CAMTA2 | 1,2 | calmodulin binding transcription activator 2 |
| 13 | RNA5SP440 | 1,1 | RNA, 5S ribosomal pseudogene 440 |
| 14 | SLC25A3 | 1,1 | solute carrier family 25 (mitochondrial carrier) |
| 15 | CNPPD1 | 1,1 | cyclin Pas1/PHO80 domain containing 1 |
| 16 | TMEM210 | 1,1 | transmembrane protein 210 |
| 17 | C6orf99 | 1,1 | chromosome 6 open reading frame 99 |
| 18 | U4atac | 1,1 | U4atac minor spliceosomal RNA |
| 19 | CTDSP2 | 1,0 | CTD (carboxy-terminal domain, RNA polymerase II, polypeptide A) small phosphatase 2 |
| 20 | CERCAM | 1,0 | cerebral endothelial cell adhesion molecule |
| 21 | CCR2 | 1,0 | chemokine (C-C motif) receptor 2 |

**Supplementary Table S5.** The up-regulated genes with the largest significant change of expression (at least 2-fold) in CD34^+^-enriched HPCs from GHD patients treated for 3 months with GH-RT compared to GHD patients before therapy

| No | Gene Symbol | log_2_(3mth GH-RT /untreated GHD) | Gene Name |
| --- | --- | --- | --- |
| 1 | SPARC | 2,32 | secreted protein, acidic, cysteine-rich (osteonectin) |
| 2 | HCAR3 | 1,99 | hydroxycarboxylic acid receptor 3 |
| 3 | IL1B | 1,72 | interleukin 1, beta |
| 4 | GCA | 1,71 | grancalcin, EF-hand calcium binding protein |
| 5 | THBS1 | 1,70 | thrombospondin 1 |
| 6 | IER3 | 1,70 | immediate early response 3 |
| 7 | ATP6V0B | 1,65 | ATPase, H+ transporting, lysosomal 21kDa, V0 subunit b |
| 8 | TNFAIP6 | 1,64 | tumor necrosis factor, alpha-induced protein 6 |
| 9 | PSMB2 | 1,64 | proteasome (prosome, macropain) subunit, beta type, 2 |
| 10 | OSM | 1,58 | oncostatin M |
| 11 | EIF4G2 | 1,57 | eukaryotic translation initiation factor 4 gamma, 2 |
| 12 | HCAR2 | 1,51 | hydroxycarboxylic acid receptor 2 |
| 13 | ARF3 | 1,48 | ADP-ribosylation factor 3 |
| 14 | SH3BGRL2 | 1,44 | SH3 domain binding glutamic acid-rich protein like 2 |
| 15 | ALPL | 1,43 | alkaline phosphatase, liver/bone/kidney |
| 16 | F13A1 | 1,43 | coagulation factor XIII, A1 polypeptide |
| 17 | IFIT2 | 1,39 | interferon-induced protein with tetratricopeptide repeats 2 |
| 18 | SOD2 | 1,39 | superoxide dismutase 2, mitochondrial |
| 19 | PGRMC1 | 1,38 | progesterone receptor membrane component 1 |
| 20 | FFAR2 | 1,37 | free fatty acid receptor 2 |
| 21 | TNFAIP3 | 1,32 | tumor necrosis factor, alpha-induced protein 3 |
| 22 | TREM1 | 1,30 | triggering receptor expressed on myeloid cells 1 |
| 23 | TNF | 1,29 | tumor necrosis factor |
| 24 | TUBB1 | 1,29 | tubulin, beta 1 class VI |
| 25 | NABP1 | 1,25 | nucleic acid binding protein 1 |
| 26 | PPBP | 1,23 | pro-platelet basic protein (chemokine (C-X-C motif) ligand 7) |
| 27 | CFD | 1,23 | complement factor D (adipsin) |
| 28 | PTGS2 | 1,23 | prostaglandin-endoperoxide synthase 2 (prostaglandin G/H synthase and cyclooxygenase) |
| 29 | KPNB1 | 1,22 | karyopherin (importin) beta 1 |
| 30 | SMNDC1 | 1,22 | survival motor neuron domain containing 1 |
| 31 | TRIB1 | 1,18 | tribbles homolog 1 (Drosophila) |
| 32 | TARDBP | 1,17 | TAR DNA binding protein |
| 33 | USP22 | 1,17 | ubiquitin specific peptidase 22 |
| 34 | HIST2H2BE | 1,16 | histone cluster 2, H2be |
| 35 | GDI2 | 1,16 | GDP dissociation inhibitor 2 |
| 36 | IGJ | 1,14 | immunoglobulin J polypeptide, linker protein for immunoglobulin alpha and mu polypeptides |
| 37 | PTX3 | 1,14 | pentraxin 3, long |
| 38 | LRRFIP1 | 1,14 | leucine rich repeat (in FLII) interacting protein 1 |
| 39 | NFKBIZ | 1,13 | nuclear factor of kappa light polypeptide gene enhancer in B-cells inhibitor, zeta |
| 40 | CCL4 | 1,11 | chemokine (C-C motif) ligand 4 |
| 41 | HNRNPA1 | 1,11 | heterogeneous nuclear ribonucleoprotein A1 |
| 42 | GATC | 1,10 | glutamyl-tRNA(Gln) amidotransferase, subunit C homolog (bacterial) |
| 43 | DDX3X | 1,09 | DEAD (Asp-Glu-Ala-Asp) box polypeptide 3, X-linked |
| 44 | BCL2A1 | 1,09 | BCL2-related protein A1 |
| 45 | G0S2 | 1,08 | G0/G1switch 2 |
| 46 | IL1RN | 1,08 | interleukin 1 receptor antagonist |
| 47 | HSP90AB1 | 1,07 | heat shock protein 90kDa alpha (cytosolic), class B member 1 |
| 48 | CLU | 1,07 | clusterin |
| 49 | STEAP4 | 1,06 | STEAP family member 4 |
| 50 | MNDA | 1,05 | myeloid cell nuclear differentiation antigen |
| 51 | SIRPB1 | 1,05 | signal-regulatory protein beta 1 |
| 52 | RALB | 1,04 | v-ral simian leukemia viral oncogene homolog B (ras related; GTP binding protein) |
| 53 | AQP9 | 1,04 | aquaporin 9 |
| 54 | GBP2 | 1,03 | guanylate binding protein 2, interferon-inducible |
| 55 | LPCAT2 | 1,03 | lysophosphatidylcholine acyltransferase 2 |
| 56 | EMR1 | 1,01 | egf-like module containing, mucin-like, hormone receptor-like 1 |
| 57 | APOBEC3A | 1,0 | apolipoprotein B mRNA editing enzyme, catalytic polypeptide-like 3A |
| 58 | NFIL3 | 1,0 | nuclear factor, interleukin 3 regulated |
| 59 | IVNS1ABP | 1,0 | influenza virus NS1A binding protein |
| 60 | SRSF9 | 1,0 | serine/arginine-rich splicing factor 9 |
| 61 | SLC25A37 | 1,0 | solute carrier family 25 (mitochondrial iron transporter), member 37 |
| 62 | PKN2 | 1,0 | protein kinase N2 |
| 63 | NRGN | 1,0 | neurogranin (protein kinase C substrate, RC3) |
| 64 | STARD7 | 1,0 | StAR-related lipid transfer (START) domain containing 7 |

**Supplementary Table S6.** The down-regulated genes with the largest significant change of expression (at least 2-fold) in CD34^+^-enriched HPCs from GHD patients treated for 3 months with GH-RT compared to GHD patients before therapy

| No | Gene Symbol | log_2_(3mth GH-RT /untreated GHD) | Gene Name |
| --- | --- | --- | --- |
| 1 | CAPNS1 | 2,01 | calpain, small subunit 1 |
| 2 | USP22 | 1,78 | ubiquitin specific peptidase 22 |
| 3 | SOWAHC | 1,56 | sosondowah ankyrin repeat domain family member C |
| 4 | C6orf99 | 1,44 | chromosome 6 open reading frame 99 |
| 5 | SNRNP200 | 1,37 | small nuclear ribonucleoprotein 200kDa (U5) |
| 6 | SART1 | 1,33 | squamous cell carcinoma antigen recognized by T cells |
| 7 | TCEB2 | 1,31 | transcription elongation factor B (SIII), polypeptide 2 (18kDa, elongin B) |
| 8 | CANX | 1,28 | calnexin |
| 9 | ANKRD36BP1 | 1,18 | ankyrin repeat domain 36B pseudogene 1 |
| 10 | HMGN3P1 | 1,15 | high mobility group nucleosomal binding domain 3 pseudogene 1 |
| 11 | U4 | 1,14 | U4 spliceosomal RNA |
| 12 | SNORD116-6 | 1,08 | small nucleolar RNA, C/D box 116-6 |
| 13 | NPPC | 1,08 | natriuretic peptide C |
| 14 | ARF3 | 1,05 | ADP-ribosylation factor 3 |
| 15 | CERCAM | 1,04 | cerebral endothelial cell adhesion molecule |
| 16 | SLC12A1 | 1,02 | solute carrier family 12 (sodium/potassium/chloride transporters), member 1 |
| 17 | CELF2-AS1 | 1,01 | CELF2 antisense RNA 1 |
| 18 | SNORA1 | 1,0 | Small nucleolar RNA SNORA1 |
| 19 | RPS24 | 1,0 | ribosomal protein S24 |
| 20 | RNA5SP116 | 1,0 | RNA, 5S ribosomal pseudogene 116 |
| 21 | POU5F1B | 1,0 | POU class 5 homeobox 1B |
| 22 | CYP2D6 | 1,0 | cytochrome P450, family 2, subfamily D, polypeptide 6 |

**Supplementary Table S7.** The up-regulated genes with the largest significant change of expression (at least 2-fold) in CD34^+^-enriched HPCs from GHD patients treated for 6 months with GH-RT compared to patients treated for 3 months with GH-RT

| No | Gene Symbol | log_2_(6mth/3mth GH-RT) | Gene Name |
| --- | --- | --- | --- |
| 1 | IFI44L | 3,06 | interferon-induced protein 44-like |
| 2 | RSAD2 | 2,66 | radical S-adenosyl methionine domain containing 2 |
| 3 | IFIT1 | 2,44 | interferon-induced protein with tetratricopeptide repeats 1 |
| 4 | CDK6 | 2,43 | cyclin-dependent kinase 6 |
| 5 | RNU5E-1 | 2,42 | RNA, U5E small nuclear 1 |
| 6 | IFI44 | 2,15 | interferon-induced protein 44 |
| 7 | MYB | 2,15 | v-myb myeloblastosis viral oncogene homolog (avian) |
| 8 | SNORA38B | 2,02 | small nucleolar RNA, H/ACA box 38B |
| 9 | CRHBP | 1,99 | corticotropin releasing hormone binding protein |
| 10 | HINT1 | 1,97 | histidine triad nucleotide binding protein 1 |
| 11 | NPM1 | 1,89 | nucleophosmin (nucleolar phosphoprotein B23, numatrin) |
| 12 | CD34 | 1,84 | CD34 molecule |
| 13 | PROM1 | 1,79 | prominin 1 |
| 14 | SERPING1 | 1,72 | serpin peptidase inhibitor, clade G (C1 inhibitor), member 1 |
| 15 | ANKRD28 | 1,70 | ankyrin repeat domain 28 |
| 16 | CPA3 | 1,62 | carboxypeptidase A3 (mast cell) |
| 17 | U1 | 1,60 | U1 spliceosomal RNA |
| 18 | IFI6 | 1,56 | interferon, alpha-inducible protein 6 |
| 19 | NRIP1 | 1,55 | nuclear receptor interacting protein 1 |
| 20 | RPS24 | 1,55 | ribosomal protein S24 |
| 21 | ANAPC5 | 1,54 | anaphase promoting complex subunit 5 |
| 22 | OR2L8 | 1,49 | olfactory receptor, family 2, subfamily L, member 8 |
| 23 | LY6E | 1,44 | lymphocyte antigen 6 complex, locus E |
| 24 | U3 | 1,44 | Small nucleolar RNA U3 |
| 25 | MX1 | 1,43 | myxovirus (influenza virus) resistance 1, interferon-inducible protein p78 |
| 26 | TESPA1 | 1,42 | thymocyte expressed, positive selection associated 1 |
| 27 | U4 | 1,39 | U4 spliceosomal RNA |
| 28 | RNU5B-1 | 1,37 | RNA, U5B small nuclear 1 |
| 29 | EIF2AK2 | 1,34 | eukaryotic translation initiation factor 2-alpha kinase 2 |
| 30 | SSBP2 | 1,32 | single-stranded DNA binding protein 2 |
| 31 | RNU1-11P | 1,31 | RNA, U1 small nuclear 11, pseudogene |
| 32 | SNORA68 | 1,31 | Small nucleolar RNA SNORA68 |
| 33 | OAS3 | 1,30 | 2'-5'-oligoadenylate synthetase 3, 100kDa |
| 34 | NPR3 | 1,28 | natriuretic peptide receptor C/guanylate cyclase C (atrionatriuretic peptide receptor C) |
| 35 | IFITM3 | 1,27 | interferon induced transmembrane protein 3 |
| 36 | IFIT3 | 1,24 | interferon-induced protein with tetratricopeptide repeats 3 |
| 37 | NLK | 1,22 | nemo-like kinase |
| 38 | OAS1 | 1,20 | 2'-5'-oligoadenylate synthetase 1, 40/46kDa |
| 39 | LAPTM4B | 1,18 | lysosomal protein transmembrane 4 beta |
| 40 | C11orf58 | 1,15 | chromosome 11 open reading frame 58 |
| 41 | ZNF521 | 1,15 | zinc finger protein 521 |
| 42 | SNORA54 | 1,14 | small nucleolar RNA, H/ACA box 54 |
| 43 | HPGDS | 1,13 | hematopoietic prostaglandin D synthase |
| 44 | ATP8B4 | 1,13 | ATPase, class I, type 8B, member 4 |
| 45 | C6orf48 | 1,12 | chromosome 6 open reading frame 48 |
| 46 | RPL11 | 1,11 | ribosomal protein L11 |
| 47 | FAM117A | 1,11 | family with sequence similarity 117, member A |
| 48 | NARS | 1,11 | asparaginyl-tRNA synthetase |
| 49 | HIST1H2BF | 1,08 | histone cluster 1, H2bf |
| 50 | RNU4-1 | 1,08 | RNA, U4 small nuclear 1 |
| 51 | RPL35 | 1,08 | ribosomal protein L35 |
| 52 | SLC10A5 | 1,08 | solute carrier family 10 (sodium/bile acid cotransporter family), member 5 |
| 53 | APEX1 | 1,07 | APEX nuclease (multifunctional DNA repair enzyme) 1 |
| 54 | ELMO1 | 1,07 | engulfment and cell motility 1 |
| 55 | ATP6V0A2 | 1,07 | ATPase, H+ transporting, lysosomal V0 subunit a2 |
| 56 | GCSAML | 1,07 | germinal center-associated, signaling and motility-like |
| 57 | MT2A | 1,06 | metallothionein 2A |
| 58 | KHDRBS1 | 1,06 | KH domain containing, RNA binding, signal transduction associated 1 |
| 59 | SNORD116-14 | 1,04 | small nucleolar RNA, C/D box 116-14 |
| 60 | OAS2 | 1,04 | 2'-5'-oligoadenylate synthetase 2, 69/71kDa |
| 61 | PRSS57 | 1,04 | protease, serine, 57 |
| 62 | SNORA42 | 1,04 | Small nucleolar RNA SNORA42/SNORA80 family |
| 63 | SLC40A1 | 1,02 | solute carrier family 40 (iron-regulated transporter), member 1 |
| 64 | HLA-DRB5 | 1,02 | major histocompatibility complex, class II, DR beta 5 |
| 65 | MEIS1 | 1,02 | Meis homeobox 1 |
| 66 | IFIT2 | 1,0 | interferon-induced protein with tetratricopeptide repeats 2 |
| 67 | XAF1 | 1,0 | XIAP associated factor 1 |
| 68 | CD164 | 1,0 | CD164 molecule, sialomucin |
| 69 | RPL28 | 1,0 | ribosomal protein L28 |
| 70 | MT1P2 | 1,0 | metallothionein 1 pseudogene 2 |
| 71 | ERMP1 | 1,0 | endoplasmic reticulum metallopeptidase 1 |

**Supplementary Table S8:** The down-regulated genes with the largest significant change of expression (at least 2-fold) in CD34^+^-enriched HPCs from GHD patients treated for 6 months with GH-RT compared to patients treated for 3 months with GH-RT

| No | Gene Symbol | log_2_(6mth/3mth GH-RT) | Gene Name |
| --- | --- | --- | --- |
| 1 | IER3 | 2,65 | immediate early response 3 |
| 2 | HCAR3 | 2,42 | hydroxycarboxylic acid receptor 3 |
| 3 | IGJ | 2,26 | immunoglobulin J polypeptide, linker protein for immunoglobulin alpha and mu polypeptides |
| 4 | F13A1 | 2,12 | coagulation factor XIII, A1 polypeptide |
| 5 | CYTIP | 1,92 | cytohesin 1 interacting protein |
| 6 | TUBB1 | 1,90 | tubulin, beta 1 class VI |
| 7 | TNF | 1,82 | tumor necrosis factor |
| 8 | PTGS2 | 1,81 | prostaglandin-endoperoxide synthase 2 (prostaglandin G/H synthase and cyclooxygenase) |
| 9 | ATP6V0B | 1,81 | ATPase, H+ transporting, lysosomal 21kDa, V0 subunit b |
| 10 | SPARC | 1,79 | secreted protein, acidic, cysteine-rich (osteonectin) |
| 11 | GNLY | 1,72 | granulysin |
| 12 | ICAM1 | 1,70 | intercellular adhesion molecule 1 |
| 13 | HCAR2 | 1,69 | hydroxycarboxylic acid receptor 2 |
| 14 | PPBP | 1,66 | pro-platelet basic protein (chemokine (C-X-C motif) ligand 7) |
| 15 | IL1B | 1,64 | interleukin 1, beta |
| 16 | ANPEP | 1,64 | alanyl (membrane) aminopeptidase |
| 17 | G0S2 | 1,62 | G0/G1switch 2 |
| 18 | CHI3L1 | 1,61 | chitinase 3-like 1 (cartilage glycoprotein-39) |
| 19 | TNFAIP3 | 1,59 | tumor necrosis factor, alpha-induced protein 3 |
| 20 | STEAP4 | 1,57 | STEAP family member 4 |
| 21 | IL8 | 1,57 | interleukin 8 |
| 22 | SLC6A6 | 1,54 | solute carrier family 6 (neurotransmitter transporter, taurine), member 6 |
| 23 | MMP9 | 1,53 | matrix metallopeptidase 9 (gelatinase B, 92kDa gelatinase, 92kDa type IV collagenase) |
| 24 | Metazoa_SRP | 1,52 | Metazoan signal recognition particle RNA |
| 25 | OSM | 1,51 | oncostatin M |
| 26 | CCL5 | 1,50 | chemokine (C-C motif) ligand 5 |
| 27 | VNN2 | 1,50 | vanin 2 |
| 28 | FLNA | 1,48 | filamin A, alpha |
| 29 | TNFAIP2 | 1,48 | tumor necrosis factor, alpha-induced protein 2 |
| 30 | TCEB2 | 1,47 | transcription elongation factor B (SIII), polypeptide 2 (18kDa, elongin B) |
| 31 | MME | 1,47 | membrane metallo-endopeptidase |
| 32 | RNF19B | 1,47 | ring finger protein 19B |
| 33 | VCAN | 1,46 | versican |
| 34 | CXCR4 | 1,44 | chemokine (C-X-C motif) receptor 4 |
| 35 | IL7R | 1,42 | interleukin 7 receptor |
| 36 | NRGN | 1,41 | neurogranin (protein kinase C substrate, RC3) |
| 37 | TNFRSF1B | 1,37 | tumor necrosis factor receptor superfamily, member 1B |
| 38 | CCL3 | 1,35 | chemokine (C-C motif) ligand 3 |
| 39 | CD14 | 1,35 | CD14 molecule |
| 40 | FAM129A | 1,34 | family with sequence similarity 129, member A |
| 41 | CXCR2 | 1,34 | chemokine (C-X-C motif) receptor 2 |
| 42 | NFKBIA | 1,34 | nuclear factor of kappa light polypeptide gene enhancer in B-cells inhibitor, alpha |
| 43 | TMEM176A | 1,32 | transmembrane protein 176A |
| 44 | PPP1R15A | 1,31 | protein phosphatase 1, regulatory subunit 15A |
| 45 | SOD2 | 1,31 | superoxide dismutase 2, mitochondrial |
| 46 | CLU | 1,29 | clusterin |
| 47 | CAPNS1 | 1,28 | calpain, small subunit 1 |
| 48 | MAST3 | 1,28 | microtubule associated serine/threonine kinase 3 |
| 49 | CFD | 1,27 | complement factor D (adipsin) |
| 50 | NFKBIZ | 1,27 | nuclear factor of kappa light polypeptide gene enhancer in B-cells inhibitor, zeta |
| 51 | IGF2R | 1,27 | insulin-like growth factor 2 receptor |
| 52 | TREM1 | 1,26 | triggering receptor expressed on myeloid cells 1 |
| 53 | COTL1 | 1,25 | coactosin-like 1 (Dictyostelium) |
| 54 | GABARAPL1 | 1,24 | GABA(A) receptor-associated protein like 1 |
| 55 | EGR1 | 1,24 | early growth response 1 |
| 56 | SLC25A37 | 1,24 | solute carrier family 25 (mitochondrial iron transporter), member 37 |
| 57 | RAF1 | 1,24 | v-raf-1 murine leukemia viral oncogene homolog 1 |
| 58 | IL13RA1 | 1,23 | interleukin 13 receptor, alpha 1 |
| 59 | ASAP1 | 1,23 | ArfGAP with SH3 domain, ankyrin repeat and PH domain 1 |
| 60 | PILRA | 1,20 | paired immunoglobin-like type 2 receptor alpha |
| 61 | GLUL | 1,20 | glutamate-ammonia ligase |
| 62 | PGRMC1 | 1,19 | progesterone receptor membrane component 1 |
| 63 | CNBP | 1,18 | CCHC-type zinc finger, nucleic acid binding protein |
| 64 | LRRK2 | 1,18 | leucine-rich repeat kinase 2 |
| 65 | KAT7 | 1,18 | K(lysine) acetyltransferase 7 |
| 66 | SIGLEC9 | 1,18 | sialic acid binding Ig-like lectin 9 |
| 67 | OGFRL1 | 1,17 | opioid growth factor receptor-like 1 |
| 68 | ZFP36 | 1,16 | zinc finger protein 36, C3H type, homolog (mouse) |
| 69 | SRSF9 | 1,16 | serine/arginine-rich splicing factor 9 |
| 70 | ECE1 | 1,16 | endothelin converting enzyme 1 |
| 71 | IQGAP1 | 1,15 | IQ motif containing GTPase activating protein 1 |
| 72 | FRAT2 | 1,14 | frequently rearranged in advanced T-cell lymphomas 2 |
| 73 | NKG7 | 1,14 | natural killer cell group 7 sequence |
| 74 | EMR3 | 1,14 | egf-like module containing, mucin-like, hormone receptor-like 3 |
| 75 | THBS1 | 1,13 | thrombospondin 1 |
| 76 | BTG2 | 1,13 | BTG family, member 2 |
| 77 | TMEM154 | 1,13 | transmembrane protein 154 |
| 78 | RBM47 | 1,13 | RNA binding motif protein 47 |
| 79 | ALPL | 1,13 | alkaline phosphatase, liver/bone/kidney |
| 80 | MYO1F | 1,13 | myosin IF |
| 81 | PRKCD | 1,12 | protein kinase C, delta |
| 82 | ZDHHC18 | 1,12 | zinc finger, DHHC-type containing 18 |
| 83 | PARP8 | 1,11 | poly (ADP-ribose) polymerase family, member 8 |
| 84 | CXCR1 | 1,10 | chemokine (C-X-C motif) receptor 1 |
| 85 | JTB | 1,09 | jumping translocation breakpoint |
| 86 | PTPRJ | 1,09 | protein tyrosine phosphatase, receptor type, J |
| 87 | IL1R2 | 1,09 | interleukin 1 receptor, type II |
| 88 | R3HDM4 | 1,09 | R3H domain containing 4 |
| 89 | ACPP | 1,08 | acid phosphatase, prostate |
| 90 | RHOB | 1,08 | ras homolog family member B |
| 91 | DYSF | 1,08 | dysferlin, limb girdle muscular dystrophy 2B (autosomal recessive) |
| 92 | OSBPL8 | 1,08 | oxysterol binding protein-like 8 |
| 93 | MMD | 1,08 | monocyte to macrophage differentiation-associated |
| 94 | LILRB2 | 1,08 | leukocyte immunoglobulin-like receptor, subfamily B (with TM and ITIM domains), member 2 |
| 95 | C10orf54 | 1,07 | chromosome 10 open reading frame 54 |
| 96 | CFP | 1,07 | complement factor properdin |
| 97 | BCL3 | 1,07 | B-cell CLL/lymphoma 3 |
| 98 | TREML1 | 1,06 | triggering receptor expressed on myeloid cells-like 1 |
| 99 | PIK3R5 | 1,06 | phosphoinositide-3-kinase, regulatory subunit 5 |
| 100 | RGS2 | 1,06 | regulator of G-protein signaling 2, 24kDa |
| 101 | NINJ1 | 1,06 | ninjurin 1 |
| 102 | NLRP12 | 1,06 | NLR family, pyrin domain containing 12 |
| 103 | CDA | 1,05 | cytidine deaminase |
| 104 | LYST | 1,05 | lysosomal trafficking regulator |
| 105 | ARAP1 | 1,05 | ArfGAP with RhoGAP domain, ankyrin repeat and PH domain 1 |
| 106 | SLC11A1 | 1,04 | solute carrier family 11 (proton-coupled divalent metal ion transporters), member 1 |
| 107 | AGTPBP1 | 1,04 | ATP/GTP binding protein 1 |
| 108 | IER2 | 1,04 | immediate early response 2 |
| 109 | CD3G | 1,04 | CD3g molecule, gamma (CD3-TCR complex) |
| 110 | CCL4 | 1,03 | chemokine (C-C motif) ligand 4 |
| 111 | BCL6 | 1,03 | B-cell CLL/lymphoma 6 |
| 112 | LRP1 | 1,03 | low density lipoprotein receptor-related protein 1 |
| 113 | TLR6 | 1,02 | toll-like receptor 6 |
| 114 | APBB1IP | 1,02 | amyloid beta (A4) precursor protein-binding, family B, member 1 interacting protein |
| 115 | CHST15 | 1,01 | carbohydrate (N-acetylgalactosamine 4-sulfate 6-O) sulfotransferase 15 |
| 116 | RASSF3 | 1,01 | Ras association (RalGDS/AF-6) domain family member 3 |
| 117 | SEC14L1 | 1,01 | SEC14-like 1 (S. cerevisiae) |
| 118 | CYP4F3 | 1,0 | cytochrome P450, family 4, subfamily F, polypeptide 3 |
| 119 | TLR2 | 1,0 | toll-like receptor 2 |
| 120 | CDC42SE1 | 1,0 | CDC42 small effector 1 |
| 121 | TRIB1 | 1,0 | tribbles homolog 1 (Drosophila) |
| 122 | PREX1 | 1,0 | phosphatidylinositol-3,4,5-trisphosphate-dependent Rac exchange factor 1 |
| 123 | ITGAM | 1,0 | integrin, alpha M (complement component 3 receptor 3 subunit) |
| 124 | IQSEC1 | 1,0 | IQ motif and Sec7 domain 1 |
| 125 | ARPC1B | 1,0 | actin related protein 2/3 complex, subunit 1B, 41kDa |
| 126 | MGAM | 1,0 | maltase-glucoamylase (alpha-glucosidase) |
| 127 | ARHGAP26 | 1,0 | Rho GTPase activating protein 26 |
| 128 | SH3BGRL2 | 1,0 | SH3 domain binding glutamic acid-rich protein like 2 |
| 129 | EFHD2 | 1,0 | EF-hand domain family, member D2 |
| 130 | ITGB2 | 1,0 | integrin, beta 2 (complement component 3 receptor 3 and 4 subunit) |
| 131 | DENND3 | 1,0 | DENN/MADD domain containing 3 |
| 132 | LILRA1 | 1,0 | leukocyte immunoglobulin-like receptor, subfamily A (with TM domain), member 1 |
| 133 | IL18RAP | 1,0 | interleukin 18 receptor accessory protein |
| 134 | SLFN5 | 1,0 | schlafen family member 5 |
| 135 | KXD1 | 1,0 | KxDL motif containing 1 |
| 136 | OGDH | 1,0 | oxoglutarate (alpha-ketoglutarate) dehydrogenase (lipoamide) |
| 137 | IL10RA | 1,0 | interleukin 10 receptor, alpha |
| 138 | JUNB | 1,0 | jun B proto-oncogene |
| 139 | FCAR | 1,0 | Fc fragment of IgA, receptor for |
| 140 | GPR97 | 1,0 | G protein-coupled receptor 97 |
| 141 | NOTCH1 | 1,0 | notch 1 |
| 142 | SLC15A3 | 1,0 | solute carrier family 15, member 3 |
| 143 | CEBPB | 1,0 | CCAAT/enhancer binding protein (C/EBP), beta |
| 144 | FAM101B | 1,0 | family with sequence similarity 101, member B |
| 145 | PI3 | 1,0 | peptidase inhibitor 3, skin-derived |
| 146 | SLCO3A1 | 1,0 | solute carrier organic anion transporter family, member 3A1 |

**Supplementary Table S9:** The up-regulated genes with the largest significant change of expression (at least 2-fold) in CD34^+^-enriched HPCs from GHD patients before therapy compared to controls

| No | Gene Symbol | log_2_(GHD/  controls) | Gene Name |
| --- | --- | --- | --- |
| 1 | SNORA38B | 1,99 | small nucleolar RNA, H/ACA box 38B |
| 2 | HLA-DPB1 | 1,55 | major histocompatibility complex, class II, DP beta 1 |
| 3 | HIST1H2BH | 1,54 | histone cluster 1, H2bh |
| 4 | TCL1A | 1,47 | T-cell leukemia/lymphoma 1A |
| 5 | MS4A1 | 1,45 | membrane-spanning 4-domains, subfamily A, member 1 |
| 6 | CD79A | 1,33 | CD79a molecule, immunoglobulin-associated alpha |
| 7 | U4 | 1,30 | U4 spliceosomal RNA |
| 8 | IGHM | 1,29 | immunoglobulin heavy constant mu |
| 9 | RNA5SP260 | 1,29 | RNA, 5S ribosomal pseudogene 260 |
| 10 | FAM86FP | 1,16 | family with sequence similarity 86, member F, |
| 11 | RPS3AP5 | 1,13 | ribosomal protein S3A pseudogene 5 |
| 12 | SNORA23 | 1,11 | small nucleolar RNA, H/ACA box 23 |
| 13 | FAM129C | 1,05 | family with sequence similarity 129, member C |
| 14 | RPL35 | 1,04 | ribosomal protein L35 |
| 15 | KARS | 1,0 | lysyl-tRNA synthetase |
| 16 | HLA-DQA1 | 1,0 | major histocompatibility complex, class II, DQ alpha 1 |

**Supplementary Table S10.** The down-regulated genes with the largest significant change of expression (at least 2-fold) in CD34^+^-enriched HPCs from GHD patients before therapy compared to controls

| No | Gene Symbol | log_2_(GHD/  controls) | Gene Name |
| --- | --- | --- | --- |
| 1 | PTGS2 | 2,42 | prostaglandin-endoperoxide synthase 2 (prostaglandin G/H synthase and cyclooxygenase) |
| 2 | PSMB2 | 2,07 | proteasome (prosome, macropain) subunit, beta type, 2 |
| 3 | FFAR2 | 2,06 | free fatty acid receptor 2 |
| 4 | SMNDC1 | 2,04 | survival motor neuron domain containing 1 |
| 5 | IL1B | 2,00 | interleukin 1, beta |
| 6 | HCAR3 | 1,99 | hydroxycarboxylic acid receptor 3 |
| 7 | ATP6V0B | 1,74 | ATPase, H+ transporting, lysosomal 21kDa, V0 subunit b |
| 8 | ALPL | 1,69 | alkaline phosphatase, liver/bone/kidney |
| 9 | EIF3D | 1,69 | eukaryotic translation initiation factor 3, subunit D |
| 10 | GCA | 1,67 | grancalcin, EF-hand calcium binding protein |
| 11 | MGAM | 1,65 | maltase-glucoamylase (alpha-glucosidase) |
| 12 | SRSF9 | 1,64 | serine/arginine-rich splicing factor 9 |
| 13 | PRPF8 | 1,63 | PRP8 pre-mRNA processing factor 8 homolog (S. cerevisiae) |
| 14 | ARF3 | 1,61 | ADP-ribosylation factor 3 |
| 15 | TRIB1 | 1,59 | tribbles homolog 1 (Drosophila) |
| 16 | IER3 | 1,59 | immediate early response 3 |
| 17 | TREM1 | 1,53 | triggering receptor expressed on myeloid cells 1 |
| 18 | BTG2 | 1,52 | BTG family, member 2 |
| 19 | SLC25A37 | 1,47 | solute carrier family 25 (mitochondrial iron transporter), member 37 |
| 20 | USP22 | 1,45 | ubiquitin specific peptidase 22 |
| 21 | CCR1 | 1,44 | chemokine (C-C motif) receptor 1 |
| 22 | ERV3-1 | 1,43 | endogenous retrovirus group 3, member 1 |
| 23 | ACSL1 | 1,43 | acyl-CoA synthetase long-chain family member 1 |
| 24 | IL2RB | 1,42 | interleukin 2 receptor, beta |
| 25 | KCNJ15 | 1,41 | potassium inwardly-rectifying channel, subfamily J, member 15 |
| 26 | NABP1 | 1,40 | nucleic acid binding protein 1 |
| 27 | SART1 | 1,40 | squamous cell carcinoma antigen recognized by T cells |
| 28 | CYTIP | 1,38 | cytohesin 1 interacting protein |
| 29 | STEAP4 | 1,37 | STEAP family member 4 |
| 30 | SOD2 | 1,36 | superoxide dismutase 2, mitochondrial |
| 31 | FPR2 | 1,35 | formyl peptide receptor 2 |
| 32 | CXCL1 | 1,33 | chemokine (C-X-C motif) ligand 1 (melanoma growth stimulating activity, alpha) |
| 33 | B4GALT5 | 1,33 | UDP-Gal:betaGlcNAc beta 1,4- galactosyltransferase, polypeptide 5 |
| 34 | KAT7 | 1,33 | K(lysine) acetyltransferase 7 |
| 35 | FAM129A | 1,31 | family with sequence similarity 129, member A |
| 36 | SLC2A3 | 1,31 | solute carrier family 2 (facilitated glucose transporter), member 3 |
| 37 | SLC11A1 | 1,29 | solute carrier family 11 (proton-coupled divalent metal ion transporters), member 1 |
| 38 | PFKFB3 | 1,28 | 6-phosphofructo-2-kinase/fructose-2,6-biphosphatase 3 |
| 39 | ZNF737 | 1,27 | zinc finger protein 737 |
| 40 | IL1R2 | 1,27 | interleukin 1 receptor, type II |
| 41 | UBE2D1 | 1,25 | ubiquitin-conjugating enzyme E2D 1 |
| 42 | CFL1 | 1,25 | cofilin 1 (non-muscle) |
| 43 | TNFAIP3 | 1,25 | tumor necrosis factor, alpha-induced protein 3 |
| 44 | BCL2A1 | 1,24 | BCL2-related protein A1 |
| 45 | AQP9 | 1,24 | aquaporin 9 |
| 46 | DYSF | 1,23 | dysferlin, limb girdle muscular dystrophy 2B (autosomal recessive) |
| 47 | PELI1 | 1,23 | pellino E3 ubiquitin protein ligase 1 |
| 48 | RGS2 | 1,22 | regulator of G-protein signaling 2, 24kDa |
| 49 | PADI2 | 1,22 | peptidyl arginine deiminase, type II |
| 50 | FAM91A1 | 1,21 | family with sequence similarity 91, member A1 |
| 51 | ILF2 | 1,21 | interleukin enhancer binding factor 2, 45kDa |
| 52 | MAPK14 | 1,20 | mitogen-activated protein kinase 14 |
| 53 | HNRNPA1 | 1,20 | heterogeneous nuclear ribonucleoprotein A1 |
| 54 | CYP27A1 | 1,20 | cytochrome P450, family 27, subfamily A, polypeptide 1 |
| 55 | CEBPD | 1,19 | CCAAT/enhancer binding protein (C/EBP), delta |
| 56 | CXCR2 | 1,19 | chemokine (C-X-C motif) receptor 2 |
| 57 | SIRPB1 | 1,19 | signal-regulatory protein beta 1 |
| 58 | STARD7 | 1,19 | StAR-related lipid transfer (START) domain containing 7 |
| 59 | MANSC1 | 1,18 | MANSC domain containing 1 |
| 60 | IL7R | 1,18 | interleukin 7 receptor |
| 61 | CTSW | 1,18 | cathepsin W |
| 62 | CST7 | 1,18 | cystatin F (leukocystatin) |
| 63 | TNFAIP6 | 1,18 | tumor necrosis factor, alpha-induced protein 6 |
| 64 | MCTP2 | 1,17 | multiple C2 domains, transmembrane 2 |
| 65 | SECTM1 | 1,17 | secreted and transmembrane 1 |
| 66 | CXCR1 | 1,17 | chemokine (C-X-C motif) receptor 1 |
| 67 | RPL28 | 1,17 | ribosomal protein L28 |
| 68 | PRF1 | 1,16 | perforin 1 (pore forming protein) |
| 69 | FPR1 | 1,14 | formyl peptide receptor 1 |
| 70 | TMEM164 | 1,12 | transmembrane protein 164 |
| 71 | ST8SIA4 | 1,12 | ST8 alpha-N-acetyl-neuraminide alpha-2,8-sialyltransferase 4 |
| 72 | FCRL6 | 1,12 | Fc receptor-like 6 |
| 73 | ST6GALNAC2 | 1,11 | ST6 (alpha-N-acetyl-neuraminyl-2,3-beta-galactosyl-1,3)-N-acetylgalactosaminide alpha-2,6-sialyltransferase 2 |
| 74 | SDCBP | 1,10 | syndecan binding protein (syntenin) |
| 75 | ATF6 | 1,10 | activating transcription factor 6 |
| 76 | CR1 | 1,10 | complement component (3b/4b) receptor 1 (Knops blood group) |
| 77 | CCDC88C | 1,09 | coiled-coil domain containing 88C |
| 78 | SSH2 | 1,09 | slingshot homolog 2 (Drosophila) |
| 79 | SNORD116-24 | 1,09 | small nucleolar RNA, C/D box 116-24 |
| 80 | CEBPB | 1,08 | CCAAT/enhancer binding protein (C/EBP), beta |
| 81 | MXD1 | 1,08 | MAX dimerization protein 1 |
| 82 | PYGL | 1,07 | phosphorylase, glycogen, liver |
| 83 | PGS1 | 1,07 | phosphatidylglycerophosphate synthase 1 |
| 84 | LRP10 | 1,07 | low density lipoprotein receptor-related protein 10 |
| 85 | BCL3 | 1,06 | B-cell CLL/lymphoma 3 |
| 86 | PLK3 | 1,06 | polo-like kinase 3 |
| 87 | TLR2 | 1,05 | toll-like receptor 2 |
| 88 | DOCK5 | 1,05 | dedicator of cytokinesis 5 |
| 89 | APBB1IP | 1,05 | amyloid beta (A4) precursor protein-binding, family B, member 1 interacting protein |
| 90 | GPR97 | 1,05 | G protein-coupled receptor 97 |
| 91 | CPQ | 1,05 | carboxypeptidase Q |
| 92 | GPR56 | 1,04 | G protein-coupled receptor 56 |
| 93 | RFWD2 | 1,04 | ring finger and WD repeat domain 2, E3 ubiquitin protein ligase |
| 94 | TSC22D3 | 1,04 | TSC22 domain family, member 3 |
| 95 | EMR2 | 1,04 | egf-like module containing, mucin-like, hormone receptor-like 2 |
| 96 | WDR26 | 1,04 | WD repeat domain 26 |
| 97 | GATC | 1,04 | glutamyl-tRNA(Gln) amidotransferase, subunit C homolog (bacterial) |
| 98 | CFLAR | 1,04 | CASP8 and FADD-like apoptosis regulator |
| 99 | IVNS1ABP | 1,03 | influenza virus NS1A binding protein |
| 100 | PPP4R1 | 1,03 | protein phosphatase 4, regulatory subunit 1 |
| 101 | HCAR2 | 1,02 | hydroxycarboxylic acid receptor 2 |
| 102 | NRD1 | 1,02 | nardilysin (N-arginine dibasic convertase) |
| 103 | UBE2R2 | 1,02 | ubiquitin-conjugating enzyme E2R 2 |
| 104 | CCL4 | 1,01 | chemokine (C-C motif) ligand 4 |
| 105 | GLT1D1 | 1,01 | glycosyltransferase 1 domain containing 1 |
| 106 | BCAP31 | 1,01 | B-cell receptor-associated protein 31 |
| 107 | TRIM25 | 1,00 | tripartite motif containing 25 |
| 108 | SART3 | 1,00 | squamous cell carcinoma antigen recognized by T cells 3 |
| 109 | BCL6 | 1,00 | B-cell CLL/lymphoma 6 |
| 110 | FCAR | 1,00 | Fc fragment of IgA, receptor for |
| 111 | LILRA5 | 1,00 | leukocyte immunoglobulin-like receptor, subfamily A (with TM domain), member 5 |
| 112 | MMP9 | 1,00 | matrix metallopeptidase 9 (gelatinase B, 92kDa gelatinase, 92kDa type IV collagenase) |
| 113 | RICTOR | 1,00 | RPTOR independent companion of MTOR, complex 2 |
| 114 | KPNB1 | 1,0 | karyopherin (importin) beta 1 |
| 115 | PROK2 | 1,0 | prokineticin 2 |
| 116 | MPP1 | 1,0 | membrane protein, palmitoylated 1, 55kDa |
| 117 | CLEC7A | 1,0 | C-type lectin domain family 7, member A |
| 118 | HCK | 1,0 | hemopoietic cell kinase |
| 119 | TMEM43 | 1,0 | transmembrane protein 43 |
| 120 | ZNF146 | 1,0 | zinc finger protein 146 |
| 121 | IGF2R | 1,0 | insulin-like growth factor 2 receptor |
| 122 | CPD | 1,0 | carboxypeptidase D |
| 123 | LITAF | 1,0 | lipopolysaccharide-induced TNF factor |
| 124 | FAM126B | 1,0 | family with sequence similarity 126, member B |
| 125 | ZFP36L1 | 1,0 | zinc finger protein 36, C3H type-like 1 |
| 126 | ITK | 1,0 | IL2-inducible T-cell kinase |
| 127 | DDX3X | 1,0 | DEAD (Asp-Glu-Ala-Asp) box polypeptide 3, X-linked |
| 128 | SLC7A7 | 1,0 | solute carrier family 7 (amino acid transporter light chain, y+L system), member 7 |
| 129 | ITGA5 | 1,0 | integrin, alpha 5 (fibronectin receptor, alpha polypeptide) |

**Supplementary Table S11.** The up-regulated genes with the largest significant change of expression (at least 2-fold) in CD34^+^-enriched HPCs from GHD patients treated for 6 months with GH-RT compared to controls

| No | Gene Symbol | log_2_(6mth GH-RT/ controls) | Gene Name |
| --- | --- | --- | --- |
| 1 | IFI44L | 3,36 | interferon-induced protein 44-like |
| 2 | SNORA38B | 3,21 | small nucleolar RNA, H/ACA box 38B |
| 3 | RSAD2 | 2,83 | radical S-adenosyl methionine domain containing 2 |
| 4 | IFIT1 | 2,58 | interferon-induced protein with tetratricopeptide repeats 1 |
| 5 | IFI44 | 2,49 | interferon-induced protein 44 |
| 6 | RNU5E-1 | 2,44 | RNA, U5E small nuclear 1 |
| 7 | MYB | 2,36 | v-myb myeloblastosis viral oncogene homolog (avian) |
| 8 | CDK6 | 2,35 | cyclin-dependent kinase 6 |
| 9 | CRHBP | 2,18 | corticotropin releasing hormone binding protein |
| 10 | CD34 | 2,17 | CD34 molecule |
| 11 | ANKRD28 | 1,99 | ankyrin repeat domain 28 |
| 12 | PROM1 | 1,88 | prominin 1 |
| 13 | CPA3 | 1,87 | carboxypeptidase A3 (mast cell) |
| 14 | IFI6 | 1,83 | interferon, alpha-inducible protein 6 |
| 15 | SNORA68 | 1,79 | Small nucleolar RNA SNORA68 |
| 16 | SNORA23 | 1,75 | small nucleolar RNA, H/ACA box 23 |
| 17 | SERPING1 | 1,75 | serpin peptidase inhibitor, clade G (C1 inhibitor), member 1 |
| 18 | U4 | 1,75 | U4 spliceosomal RNA |
| 19 | HIST1H2BH | 1,66 | histone cluster 1, H2bh |
| 20 | ANAPC5 | 1,64 | anaphase promoting complex subunit 5 |
| 21 | HIST1H2BF | 1,60 | histone cluster 1, H2bf |
| 22 | U1 | 1,59 | U1 spliceosomal RNA |
| 23 | LAPTM4B | 1,57 | lysosomal protein transmembrane 4 beta |
| 24 | OR2L8 | 1,57 | olfactory receptor, family 2, subfamily L, member 8 |
| 25 | NRIP1 | 1,55 | nuclear receptor interacting protein 1 |
| 26 | NPM1 | 1,53 | nucleophosmin (nucleolar phosphoprotein B23, numatrin) |
| 27 | RPL35 | 1,53 | ribosomal protein L35 |
| 28 | IFIT2 | 1,53 | interferon-induced protein with tetratricopeptide repeats 2 |
| 29 | U6 | 1,53 | U6 spliceosomal RNA |
| 30 | XAF1 | 1,52 | XIAP associated factor 1 |
| 31 | HINT1 | 1,49 | histidine triad nucleotide binding protein 1 |
| 32 | SCARNA13 | 1,44 | small Cajal body-specific RNA 13 |
| 33 | RNU5B-1 | 1,42 | RNA, U5B small nuclear 1 |
| 34 | SAMD9L | 1,41 | sterile alpha motif domain containing 9-like |
| 35 | SSBP2 | 1,41 | single-stranded DNA binding protein 2 |
| 36 | NARS | 1,40 | asparaginyl-tRNA synthetase |
| 37 | HIST1H3I | 1,40 | histone cluster 1, H3i |
| 38 | EIF2AK2 | 1,40 | eukaryotic translation initiation factor 2-alpha kinase 2 |
| 39 | SNORA42 | 1,38 | Small nucleolar RNA SNORA42/SNORA80 family |
| 40 | IFIT3 | 1,36 | interferon-induced protein with tetratricopeptide repeats 3 |
| 41 | OAS3 | 1,34 | 2'-5'-oligoadenylate synthetase 3, 100kDa |
| 42 | RNU5A-1 | 1,33 | RNA, U5A small nuclear 1 |
| 43 | SNORA54 | 1,28 | small nucleolar RNA, H/ACA box 54 |
| 44 | IFITM3 | 1,28 | interferon induced transmembrane protein 3 |
| 45 | MT2A | 1,27 | metallothionein 2A |
| 46 | RNA5SP161 | 1,27 | RNA, 5S ribosomal pseudogene 161 |
| 47 | MX1 | 1,27 | myxovirus (influenza virus) resistance 1, interferon-inducible protein p78 (mouse) |
| 48 | MTND6P4 | 1,26 | MT-ND6 pseudogene 4 |
| 49 | SLC40A1 | 1,25 | solute carrier family 40 (iron-regulated transporter), member 1 |
| 50 | HLA-DPB1 | 1,24 | major histocompatibility complex, class II, DP beta 1 |
| 51 | OAS1 | 1,22 | 2'-5'-oligoadenylate synthetase 1, 40/46kDa |
| 52 | ATP8B4 | 1,22 | ATPase, class I, type 8B, member 4 |
| 53 | ZNF521 | 1,21 | zinc finger protein 521 |
| 54 | PRSS57 | 1,20 | protease, serine, 57 |
| 55 | U3 | 1,18 | Small nucleolar RNA U3 |
| 56 | RPS3AP5 | 1,18 | ribosomal protein S3A pseudogene 5 |
| 57 | H1F0 | 1,17 | H1 histone family, member 0 |
| 58 | SNORA71D | 1,16 | small nucleolar RNA, H/ACA box 71D |
| 59 | NLK | 1,16 | nemo-like kinase |
| 60 | NPR3 | 1,16 | natriuretic peptide receptor C/guanylate cyclase C (atrionatriuretic peptide receptor C) |
| 61 | CLC | 1,13 | Charcot-Leyden crystal protein |
| 62 | HOXA6 | 1,13 | homeobox A6 |
| 63 | RPPH1 | 1,12 | ribonuclease P RNA component H1 |
| 64 | RNF141 | 1,11 | ring finger protein 141 |
| 65 | SLC10A5 | 1,09 | solute carrier family 10 (sodium/bile acid cotransporter family), member 5 |
| 66 | TCF4 | 1,09 | transcription factor 4 |
| 67 | RNU1-11P | 1,09 | RNA, U1 small nuclear 11, pseudogene |
| 68 | HPGDS | 1,09 | hematopoietic prostaglandin D synthase |
| 69 | U8 | 1,09 | U8 small nucleolar RNA |
| 70 | C22orf28 | 1,08 | chromosome 22 open reading frame 28 |
| 71 | MEIS1 | 1,08 | Meis homeobox 1 |
| 72 | MUC12 | 1,07 | mucin 12, cell surface associated |
| 73 | TNFAIP6 | 1,07 | tumor necrosis factor, alpha-induced protein 6 |
| 74 | TM7SF3 | 1,07 | transmembrane 7 superfamily member 3 |
| 75 | CDK2AP1 | 1,06 | cyclin-dependent kinase 2 associated protein 1 |
| 76 | GCSAML | 1,05 | germinal center-associated, signaling and motility-like |
| 77 | MT1P2 | 1,04 | metallothionein 1 pseudogene 2 |
| 78 | RPL10 | 1,03 | ribosomal protein L10 |
| 79 | ADAM28 | 1,02 | ADAM metallopeptidase domain 28 |
| 80 | RNA5SP46 | 1,01 | RNA, 5S ribosomal pseudogene 46 |
| 81 | P2RY14 | 1,01 | purinergic receptor P2Y, G-protein coupled, 14 |
| 82 | CMPK2 | 1,01 | cytidine monophosphate (UMP-CMP) kinase 2, mitochondrial |
| 83 | LY6E | 1,0 | lymphocyte antigen 6 complex, locus E |
| 84 | C1orf186 | 1,0 | chromosome 1 open reading frame 186 |
| 85 | HIST1H2BM | 1,0 | histone cluster 1, H2bm |
| 86 | SPIN1 | 1,0 | spindlin 1 |
| 87 | ATP6V0A2 | 1,0 | ATPase, H+ transporting, lysosomal V0 subunit a2 |
| 88 | OAS2 | 1,0 | 2'-5'-oligoadenylate synthetase 2, 69/71kDa |
| 89 | RNA5SP382 | 1,0 | RNA, 5S ribosomal pseudogene 382 |
| 90 | APEX1 | 1,0 | APEX nuclease (multifunctional DNA repair enzyme) 1 |

**Supplementary Table S12.** The down-regulated genes with the largest significant change of expression (at least 2-fold) in CD34^+^-enriched HPCs from GHD patients treated for 6 months with GH-RT compared to controls

| No | Gene Symbol | log_2_(6mth GH-RT/ controls) | Gene Name |
| --- | --- | --- | --- |
| 1 | CYTIP | 3,07 | cytohesin 1 interacting protein |
| 2 | PTGS2 | 3,00 | prostaglandin-endoperoxide synthase 2 (prostaglandin G/H synthase and cyclooxygenase) |
| 3 | IL2RB | 2,60 | interleukin 2 receptor, beta |
| 4 | IER3 | 2,54 | immediate early response 3 |
| 5 | HCAR3 | 2,41 | hydroxycarboxylic acid receptor 3 |
| 6 | CTSW | 2,39 | cathepsin W |
| 7 | ANPEP | 2,31 | alanyl (membrane) aminopeptidase |
| 8 | PRF1 | 2,30 | perforin 1 (pore forming protein) |
| 9 | NKG7 | 2,26 | natural killer cell group 7 sequence |
| 10 | GNLY | 2,21 | granulysin |
| 11 | ITK | 2,18 | IL2-inducible T-cell kinase |
| 12 | IL7R | 2,12 | interleukin 7 receptor |
| 13 | TCEB2 | 2,11 | transcription elongation factor B (SIII), polypeptide 2 (18kDa, elongin B) |
| 14 | MMP9 | 2,04 | matrix metallopeptidase 9 (gelatinase B, 92kDa gelatinase, 92kDa type IV collagenase) |
| 15 | IL1B | 1,93 | interleukin 1, beta |
| 16 | DENND2D | 1,89 | DENN/MADD domain containing 2D |
| 17 | CXCR4 | 1,87 | chemokine (C-X-C motif) receptor 4 |
| 18 | BTG2 | 1,85 | BTG family, member 2 |
| 19 | CD3G | 1,84 | CD3g molecule, gamma (CD3-TCR complex) |
| 20 | FCRL6 | 1,82 | Fc receptor-like 6 |
| 21 | SRSF9 | 1,81 | serine/arginine-rich splicing factor 9 |
| 22 | MGAM | 1,80 | maltase-glucoamylase (alpha-glucosidase) |
| 23 | CXCR2 | 1,80 | chemokine (C-X-C motif) receptor 2 |
| 24 | CCL5 | 1,77 | chemokine (C-C motif) ligand 5 |
| 25 | LEF1 | 1,77 | lymphoid enhancer-binding factor 1 |
| 26 | IL1R2 | 1,74 | interleukin 1 receptor, type II |
| 27 | MAST3 | 1,73 | microtubule associated serine/threonine kinase 3 |
| 28 | SLC6A6 | 1,72 | solute carrier family 6 (neurotransmitter transporter), member 6 |
| 29 | FLNA | 1,70 | filamin A, alpha |
| 30 | CAPNS1 | 1,63 | calpain, small subunit 1 |
| 31 | TNFRSF1B | 1,59 | tumor necrosis factor receptor superfamily, member 1B |
| 32 | TNFAIP2 | 1,58 | tumor necrosis factor, alpha-induced protein 2 |
| 33 | CST7 | 1,55 | cystatin F (leukocystatin) |
| 34 | CYP27A1 | 1,53 | cytochrome P450, family 27, subfamily A, polypeptide 1 |
| 35 | PRKCH | 1,48 | protein kinase C, eta |
| 36 | CD5 | 1,46 | CD5 molecule |
| 37 | CD8A | 1,40 | CD8a molecule |
| 38 | CCR7 | 1,37 | chemokine (C-C motif) receptor 7 |
| 39 | CHI3L1 | 1,23 | chitinase 3-like 1 (cartilage glycoprotein-39) |
| 40 | GPR56 | 1,18 | G protein-coupled receptor 56 |
| 41 | ILF2 | 1,12 | interleukin enhancer binding factor 2, 45kDa |
| 42 | SMNDC1 | 1,10 | survival motor neuron domain containing 1 |
| 43 | F13A1 | 1,03 | coagulation factor XIII, A1 polypeptide |
| 44 | PRPF8 | 1,01 | PRP8 pre-mRNA processing factor 8 homolog |
| 45 | GCA | 1,0 | grancalcin, EF-hand calcium binding protein |

**Supplementary Table S13.** The up-regulated genes with the largest significant change of expression (at least 2-fold) in CD34^+^-enriched HPCs from GHD patients treated for 3 months with GH-RT compared to controls

| No | Gene Symbol | log_2_(3mth GH-RT/ controls) | Gene Name |
| --- | --- | --- | --- |
| 1 | SPARC | 2,33 | secreted protein, acidic, cysteine-rich (osteonectin) |
| 2 | IGJ | 1,98 | immunoglobulin J polypeptide, linker protein for immunoglobulin alpha and mu polypeptides |
| 3 | TUBB1 | 1,65 | tubulin, beta 1 class VI |
| 4 | PGRMC1 | 1,49 | progesterone receptor membrane component 1 |
| 5 | MS4A1 | 1,39 | membrane-spanning 4-domains, subfamily A, member 1 |
| 6 | CFD | 1,28 | complement factor D (adipsin) |
| 7 | IGHM | 1,24 | immunoglobulin heavy constant mu |
| 8 | RPL10 | 1,23 | ribosomal protein L10 |
| 9 | HIST1H2BH | 1,21 | histone cluster 1, H2bh |
| 10 | SNORA38B | 1,20 | small nucleolar RNA, H/ACA box 38B |
| 11 | MMD | 1,17 | monocyte to macrophage differentiation-associated |
| 12 | RNA5SP260 | 1,16 | RNA, 5S ribosomal pseudogene 260 |
| 13 | SNORA23 | 1,11 | small nucleolar RNA, H/ACA box 23 |
| 14 | F13A1 | 1,10 | coagulation factor XIII, A1 polypeptide |
| 15 | THBS1 | 1,09 | thrombospondin 1 |
| 16 | HLA-DPB1 | 1,09 | major histocompatibility complex, class II, DP beta 1 |
| 17 | TARDBP | 1,05 | TAR DNA binding protein |
| 18 | MTND6P4 | 1,02 | MT-ND6 pseudogene 4 |
| 19 | CLU | 1,02 | clusterin |
| 20 | SH3BGRL2 | 1,01 | SH3 domain binding glutamic acid-rich protein like 2 |
| 21 | OSM | 1,0 | oncostatin M |

**Supplementary Table S14.** The down-regulated genes with the largest significant change of expression (at least 2-fold) in CD34^+^-enriched HPCs from GHD patients treated for 3 months with GH-RT compared to controls

| No | Gene Symbol | log_2_(3mth GH-RT/ controls) | Gene Name |
| --- | --- | --- | --- |
| 1 | CTSW | 1,73 | cathepsin W |
| 2 | IL2RB | 1,68 | interleukin 2 receptor, beta |
| 3 | PRPF8 | 1,58 | PRP8 pre-mRNA processing factor 8 homolog |
| 4 | ZNF146 | 1,56 | zinc finger protein 146 |
| 5 | GPR56 | 1,47 | G protein-coupled receptor 56 |
| 6 | ITK | 1,45 | IL2-inducible T-cell kinase |
| 7 | PRF1 | 1,41 | perforin 1 (pore forming protein) |
| 8 | CST7 | 1,27 | cystatin F (leukocystatin) |
| 9 | RPS24 | 1,26 | ribosomal protein S24 |
| 10 | FCRL6 | 1,22 | Fc receptor-like 6 |
| 11 | PTGS2 | 1,19 | prostaglandin-endoperoxide synthase 2 (prostaglandin G/H synthase and cyclooxygenase) |
| 12 | CD8A | 1,17 | CD8a molecule |
| 13 | CYTIP | 1,16 | cytohesin 1 interacting protein |
| 14 | CCR1 | 1,15 | chemokine (C-C motif) receptor 1 |
| 15 | YY1 | 1,12 | YY1 transcription factor |
| 16 | NKG7 | 1,12 | natural killer cell group 7 sequence |
| 17 | DENND2D | 1,07 | DENN/MADD domain containing 2D |
| 18 | CD5 | 1,05 | CD5 molecule |
| 19 | ILF2 | 1,04 | interleukin enhancer binding factor 2, 45kDa |
| 20 | HLA-DQB1 | 1,01 | major histocompatibility complex, class II, DQ beta 1 |
| 21 | CYP27A1 | 1,01 | cytochrome P450, family 27, subfamily A, polypeptide 1 |
| 22 | CCR7 | 1,01 | chemokine (C-C motif) receptor 7 |
| 23 | PRKCH | 1,0 | protein kinase C, eta |
| 24 | FKBP5 | 1,0 | FK506 binding protein 5 |
| 25 | LEF1 | 1,0 | lymphoid enhancer-binding factor 1 |
| 26 | SNRNP200 | 1,0 | small nuclear ribonucleoprotein 200kDa (U5) |
